# Exploring the clinical utility of postural outcome tools for back and neck pain clinical outcomes: a systematic scoping review

**DOI:** 10.12688/f1000research.160172.1

**Published:** 2025-01-08

**Authors:** Chinonso N Igwesi-Chidobe, Esther U Anih, Grace N Emmanuel, Benjamin C Ozumba

**Affiliations:** 1 University of Bradford Faculty of Health Studies, Bradford, England, UK; 2 University of Nigeria - Enugu Campus, Enugu, Enugu, Nigeria

**Keywords:** Outcome measures/tools, Clinical utility, Posture, Postural outcome measures, Spinal pain, back pain

## Abstract

The role of posture in spinal pain is unclear which might be linked to characteristics of postural outcome measures. This systematic scoping review mapped the clinical utility of postural outcome tools for spinal pain. Following Joanna Briggs Institute framework, twelve bibliographic databases were searched until 8 ^th^ August 2023. Article selection, characterisation/mapping and synthesis using qualitative content analysis were performed by two independent reviewers. Clinical utility was defined by psychometric and clinimetric criteria. 85 eligible studies were identified from 89 publications. Twenty-eight distinct postural outcome tools plus bespoke measures were identified. Most tools were sophisticated computer-based electronic devices or complex time-consuming questionnaires, with limited applicability in non-occupational settings. Clinical utility domains most achieved were construct validity and inter/intra-rater reliability. Tools being underpinned by the biopsychosocial model of pain, sensitivity to clinical change, and predictive validity were the least achieved. Tools had limited clinical utility and were based on postural-structural-biomechanical pain model.

## Practitioner summary

The unclear clinical relevance of posture in spinal pain may be linked to postural measurement. This scoping review mapped the clinical utility of postural outcome tools in spinal pain. Tools had limited clinical utility and were underpinned by postural-structural-biomechanical pain model rather than the biopsychosocial model underpinning spinal pain management.

## Introduction

Globally, back and neck pain are leading causes of disability-adjusted life-years (DALYs) in the working population. ^1^ The role of postural factors in first onset of pain and perpetuation of symptoms and disability are unclear and conflicting.

Systematic reviews of longitudinal studies have found that increased and prolonged trunk flexion and twisting were associated with first onset low back pain (LBP). ^2– 7^ However, different thresholds were used in quantifying postural exposure which may explain the conflicting dose-response relationships. Prolonged kneeling or squatting were more likely to predict new onset LBP in newly employed workers, ^8^ but the definition of prolonged duration appeared arbitrary and may have varying impact in different individuals. Bending and twisting were found to be associated with adverse employment outcomes such as leaving jobs and the inability to carry out normal duties, ^9^ and may affect functional health in retirement. ^10^

Inappropriate home postural habits (e.g., slumped sitting) were associated with acute LBP amongst adolescents in a cross-sectional study. ^11^ However, majority of the adolescents frequently changed their position and did not have a preferred position. ^11^ These results aligned with another cross-sectional study which found that sitting posture with increased kyphosis with crossed legs was associated with acute LBP; whilst sitting posture with upper back support and lumbar strain, with the feet supported on the floor was positively associated with chronic LBP. ^12^ Duration of computer use was associated with acute and chronic LBP whilst supporting the hip and thigh in the seat were negatively associated with acute LBP. ^12^

A systematic review with meta-analysis of cross-sectional studies between 2009-2017 found that adults with neck pain have increased forward head posture compared with asymptomatic adults and that forward head posture is associated with neck pain outcomes in adults but not in adolescents. ^13^ However, the direction of relationships is unclear, and posture might have been a consequence rather than a cause of neck pain. Unclear associations between posture and back pain was found in another cross-sectional study which found that neither children without back pain nor children with back pain had normal lumbar kyphosis or normal lumbosacral angle according to normality reference values. ^14^ These findings suggest that normality reference values may be different in diverse people and that postural factors may be less important before adulthood. In concurrence, a cross-sectional study that acknowledged neck posture clusters found that none of the four distinct clusters of sitting neck posture (upright, intermediate, slumped thorax/forward head, and erect thorax/forward head) were associated with the odds of having neck pain amongst adolescents. ^15^

Some authors in high income countries have disputed the relevance of postural factors in predicting spinal pain outcomes due to lack of clear links to changes in anatomical structures. ^16^ Other authors have concluded that any associations between posture and spinal pain outcomes are at best meagre, and that the direction of relationship is likely consequential rather than causative. ^17^ Our previous population-based cross-sectional studies in Nigeria showed no clear associations between postural factors, and back and neck pain outcomes, although results might have been due to the way postural factors were measured and analysed. ^18– 20^ A cross-sectional study in Australia found significant differences in the perception of ‘good’ posture between people with and without postural neck pain but there was no difference between the habitual posture they adopted. ^21^ The people with neck pain were individuals who perceived good neck posture as one with increased head protraction, ^21^ possibly suggesting some postural control issues.

The impact of postural interventions might help to clarify the role of postural factors on back and neck pain outcomes. Unfortunately, the effectiveness of postural interventions in preventing or treating spinal pain is equivocal. A systematic review found limited and inconsistent evidence for the effectiveness of screening tools and the interventions guided by these tools in preventing musculoskeletal injuries, or reducing musculoskeletal discomfort, work absence, claims costs and health resource utilization, and improving safe workplace behaviour and self-rated health status. ^22^ Notably, the studies included in this systematic review used different purpose-built non-validated outcome tools. ^22^ In contrast, a recent high quality 3-arm, parallel-group cluster randomised controlled trial (RCT) that adjusted for biopsychosocial factors found that interventions that increased either active breaks or postural shifts reduced new onset of neck and back pain among high-risk office workers. ^23^ Although this trial was placebo-controlled and active break and postural shifts were measured, the analyses did not link the reduction in new onset of neck and back pain to changes in postural factors making it difficult to pinpoint the contribution of postural factors to prevention of neck and back pain. ^23^

Cognitive functional therapy (CFT) is a flexible and integrated biopsychosocial behavioural approach for individualizing the management of disabling LBP. ^24^ CFT targets biopsychosocial factors such as external physical factors, internal physical factors, psychological factors, social factors, and lifestyle factors. The external physical factors include the levels and patterns of spinal loading, and awkward bending and twisting during daily life activities. ^24^ CFT acknowledges “central” (central nervous system amplifies pain or perceives non-painful stimuli as painful), “peripheral” (“mechanical stimulus–pain response” i.e. pain is momentarily provoked and relieved by specific spinal postures, movements and activities) or mixed (central sensitisation subsequently facilitates posture and movements that aggravate pain) pain mechanisms. These pain mechanisms are then targeted and corrected in a reflective rather than a prescriptive process in the CFT. ^24^ However, there is no evidence that a reduction in the exposure to postural risk factors which is targeted by the CFT contributed to the improvement in clinical outcomes associated with the CFT. This is because changes in the exposure to postural risk factors were either not measured ^25– 31^ or there were no changes in posture before and after CFT despite improvements in clinical outcomes. ^32^

The evidence for the effectiveness of other postural rehabilitation programmes is also conflicting. Postural rehabilitation exercise programmes were effective in reducing pain intensity and disability which could not be linked to improvements in posture as no postural outcome was measured. ^33^ Pilates exercise programme which targeted static posture and postural habits reduced severity of temporomandibular pain but had no effect on posture, postural habits, neck and back pain in young women with temporomandibular dysfunction. ^34^ A pretest-posttest study investigated the effect of a 4-week self-stretching exercises on postural improvements in patients with chronic neck pain caused by forward head posture and found that this reduced muscle activity and changed neck alignment. However, the definition of optimal neck posture was unclear and could not be linked to the improved outcomes reported in that study. ^35^ More recent research has been moving towards postural awareness ^36, 37^ or postural balance, postural realignment and rebalancing rather than specific ‘good’ or ‘bad’ postures. ^38, 39^ A study defined postural impairment as impaired proprioception without visual feedback. ^38^

The overall conflicting evidence regarding the role of postural factors in spinal pain may be suggesting that there are no universal ‘good’ and ‘bad’ postures for back and neck pain as these may be different in different people. Moreover, posture may not even be an aggravating factor in some people. The evidence may also be suggesting that different postural habits may be associated with new onset back and neck pain, or aggravation of existing spinal pain in different ways in different people. Therefore, evidence-based postural outcome tools could focus on individuals being sensitive to their bodies to identify aggravating postures, and aggravating duration of sustained static positions, how much time is spent in regular movement and adoption of individually relieving postures and movement strategies as self-management strategies whilst acknowledging relevant biopsychosocial factors. This aligns with the evidence-based biopsychosocial model of spinal pain ^40– 43^ and the evidence-based recommendations for symptomatic relief during spinal pain episodes to increase participation in social, occupational, and recreational activities. ^44– 46^ It is unclear to what extent existing postural outcome tools align with these evidence-based recommendations, which might explain the conflicting findings regarding the role of postural factors in back and neck pain clinical outcomes.

This systematic scoping review aimed to map and summarise the clinical utility of existing outcome tools for measuring exposure to postural risk factors for back and neck pain clinical outcomes. These clinical outcomes included but were not limited to first onset of back and/or neck pain, chronicity/perpetuation of symptoms, pain including the nature and intensity, disability etc.

## Methods

This study followed the Joanna Briggs Institute (JBI) Manual for Evidence Synthesis (last update on 27 July 2023) procedures for scoping reviews ^47^ which is detailed below. This paper is reported according to the Preferred Reporting Items for Systematic Reviews and Meta-analyses (PRISMA) Extension for Scoping Reviews (PRISMA-ScR) guidelines. ^48^

### Protocol and registration

This study was initially planned to be a systematic review and registered with the International Prospective Register of Systematic Reviews (PROSPERO CRD42022321427). The study protocol was subsequently modified following extensive internal and external discussions which highlighted the fact that the objectives of this study better aligned with a scoping review methodology. ^49, 50^

### Eligibility criteria

**Figure 1.**
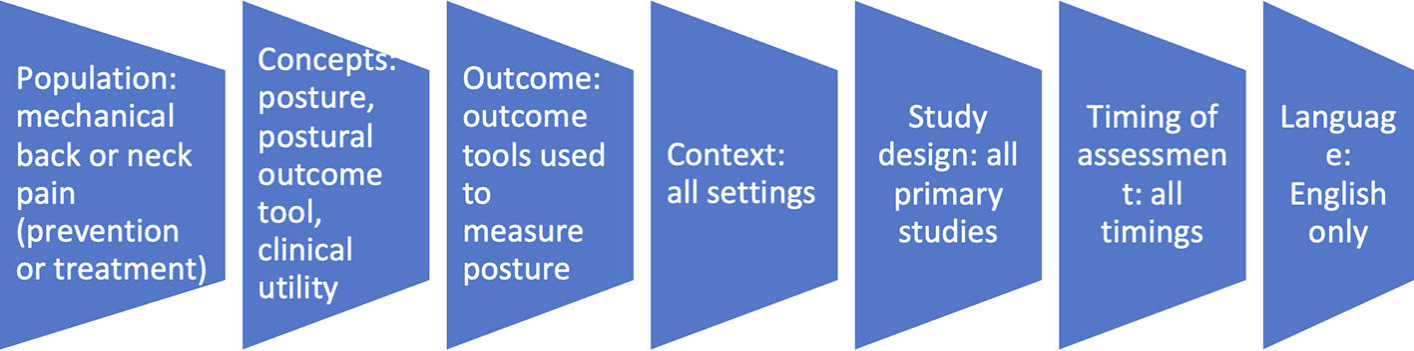
Eligibility criteria.

In summary, published full articles of primary studies that used postural outcome tools targeted at preventing first onset of mechanical back or neck pain in people without back or neck pain, or at improving clinical outcomes amongst people who already have mechanical back and/or neck pain were included. Unpublished primary studies, studies of people whose back or neck pain were due to underlying serious pathology, studies without primary data or not published in English were excluded. The illustration in Figure 1 summarises the eligibility criteria.

Inclusion criteria

1. Population: All primary studies with human participants of all ages and gender with or without mechanical back and/or neck pain but with exposure to the postural risk factors for mechanical back and neck pain clinical outcomes. In populations without back and/or neck pain, the studies were aimed at preventing back and/or neck pain. Studies involving participants with back pain or neck pain were deemed eligible due to the nature of these conditions. As highlighted in the introduction, similar biopsychosocial factors are associated with first onset of back or neck pain, and subsequent transition to chronic back or neck pain and disability. Furthermore, these factors are the focus of interventions aimed at either preventing the first onset of back or neck pain, or to reduce chronicity and disability in people who already have back or neck pain.
2. Concepts: For this study, posture was defined as the alignment or orientation of the body segments in space either whilst static such as standing, sitting, or lying down or during movement such as walking, running, or bending over. ^51, 52^ Postural outcome tool was defined as any tool or procedure that was utilised to measure or assess posture. The definition of clinical utility for this study was informed by a review of the psychometric and clinimetric criteria. ^53– 56^ Clinimetrics is the science of clinical measurements which is related to the identification of a clinical disorder and its causal variables, the tracing of the progression of the condition, and calculating its impact. Clinimetrics is a clinically based evaluation method whilst psychometrics is concerned with the theory and general techniques of measurement. ^56, 57^ The clinimetric and the psychometric approach are not contradictory, but serve different purposes, and emphasize different things. The clinimetric approach is required for the development of instruments that measure multiple constructs with a single index as observed in general clinical practice. In contrast, the psychometric approach is necessary to develop instruments that measure a single construct using multiple items which is common in psychology. The multiple constructs measured by the clinimetric approach usually include the causal variables associated with a health condition. In contrast, the single construct measured by the psychometric approach are usually indicator variables that correlate with the underlying construct to be measured but may not have causal relationships with this construct. Another difference is that clinimetrics is more content driven (with more focus on patient and clinician input or perspectives) whilst psychometrics is more statistically driven. In contrast to measures also informed by the clinimetric criteria, tools that exclusively focus on the psychometric criteria may have limited clinical applicability due to associated item redundancy where multiple items measure a single construct combined with increased length of administration. ^56^ Limited attention to clinical utility and sensitivity in the clinical environment are other limitations of exclusively using the psychometric criteria. ^56^ However, in terms of validity, reproducibility, or responsiveness, the differences between clinimetric and psychometric approaches become less obvious. ^58^ For this review, the concept of clinical utility was defined as the extent to which the postural outcome tools can detect or predict symptom changes and treatment outcomes (construct validity, predictive validity, intra and inter rater reliability, sensitivity to change); the level of ease with which the tools can be potentially applied in clinical settings for practice or research (the ease of use – if training is required to use the tool and if specialist equipment required), the ability of the tool to facilitate clinician-patient interaction and collaboration (sensibility); and the format of the tool e.g., length of assessment, wording of items, and response format or calibration of items. ^56, 59, 60^ Furthermore, as the biopsychosocial model which acknowledges that cognitive, emotional, psychological, behavioural, physical and social factors interact to perpetuate pain, ^18, 61, 62^ is the evidence-based approach underpinning current spinal pain management, the model underpinning each postural outcome tool (postural-structural-biomechanical model of pain based on the assumption that there are specific correct and wrong postures, or a biopsychosocial model of pain based on the assumption that postural aggravating factors are different in different individuals and may require individuals to identify what these are and actively modify exposure to identified aggravating postural factors) ^24– 26^ was also included within the definition of clinical utility. Construct validity was defined as the extent to which an instrument measures the construct it was intended to measure. ^63, 64^ Predictive validity was defined as the ability of the outcome tool to predict a longitudinal response to treatment and clinical outcomes. ^56^ Sensitivity to change was defined as the ability of a measure to detect subtle change and thereby discriminate patient groups, detect treatment effects and differentiate active treatment effects from placebo (in contrast to responsiveness which is the ability of an instrument to detect change over time). ^56^
3. Outcomes: All outcome tools and methods utilised to measure posture amongst people with back and/or neck pain (studies measuring treatment effects) and amongst people without back and/or neck pain (studies measuring the success of preventing back or neck pain) were eligible.
4. Context: There was no restriction based on the setting for the application of the tools in the primary studies. All settings (clinical, school, occupational, community settings etc.) were eligible for inclusion.
5. Study design: All primary studies that involved measurements using any postural outcome tool or method for people with back and/or neck pain were eligible for inclusion. Studies aimed at preventing back and neck pain in individuals without existing spinal pain using postural interventions are eligible for inclusion if they utilised a postural outcome tool for the cervical and/or lumbar spinal regions.
6. Timing of assessment: There were no restrictions based on the timing of postural assessment.
7. Language: Only studies published in English were eligible due to cost restrictions.

Exclusion criteria

Studies in which all the included participants had back and/or neck pain which was due to serious underlying pathology such as radiculopathy, spinal stenosis, ankylosing spondylitis, rheumatoid arthritis or other inflammatory diseases, cauda equina syndrome, progressive paresis, fracture, suspected tumour, or local infection were excluded. Studies without a full report such as those published only as abstracts, and unpublished studies including those published in research repositories without peer review were excluded. Studies published as abstracts were excluded because of limited reported data regarding clinical utility and study characteristics. Exclusion of unpublished studies was to ensure that the postural outcome tools were already available for use and that the clinical utility data reported were peer reviewed.

### Information sources

PubMed, CINAHL, CENTRAL, Global Index Medicus, African Index Medicus, African Journal Online, WPRIM (Western Pacific Region Index Medicus), LILACS (Latin American and Caribean Centre on Health Science Information), IMEMR (Index Medicus for South-East Asia Region), IRIS (WHO digital publications), and BLDS (British Library for Development Studies) were searched from inception up to 8 ^th^ August 2023. Additional studies were retrieved from Google scholar and the reference list of relevant studies including systematic reviews.

### Search strategy

The search strategies for the databases were informed by the Cochrane handbook, ^65, 66^ JBI framework, ^49, 50^ and PRISMA 2020 guidelines. ^67, 68^ The searches involved the combination of MeSH and free text terms and word variants for posture, spinal pain, back and neck pain, postural interventions, and outcome measurement. The search strategies (published as extended data) ^69^ were piloted and adapted to improve sensitivity and specificity before final use.

### Selection of sources of evidence

Screening of articles for eligibility was done in two phases. The first phase involved EUA and GNE independently screening the titles and abstracts of published papers for those that meet the inclusion criteria. Papers that met the eligibility criteria at this stage were moved to the second phase of screening. Disagreements at this stage were resolved by moving those papers to the second phase of screening. The second phase of screening involved EUA and GNE independently reading through the full texts of articles to confirm their eligibility. Disagreements regarding the inclusion of a study at this stage were resolved by discussions with CNI-C. Details of the study process is illustrated in a flow chart ( Figure 3).

### Data items and data charting process

**Figure 2.**
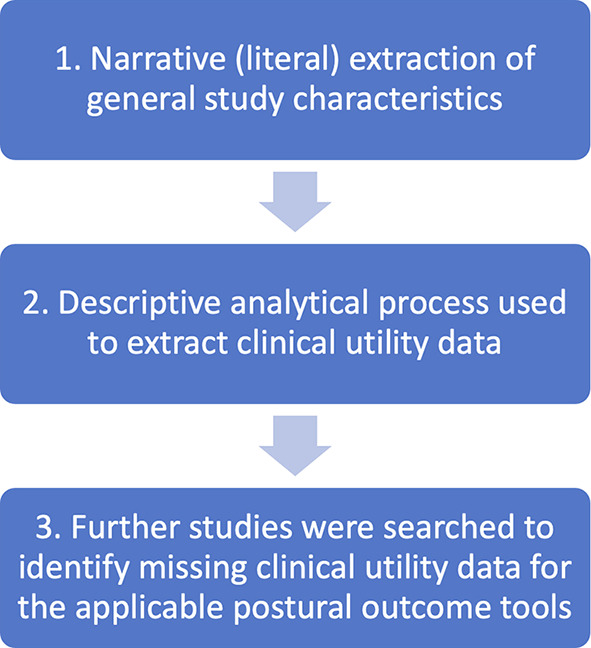
Data charting process.

CNI-C developed the data charting form. CNI-C and EUA piloted the data charting form by independently extracting data from a random sample of studies including three observational studies and three intervention studies. CNI-C and EUA discussed inconsistencies in extracted data and improvements were subsequently made to the form to align with the objectives of the study. Data charting is the process of data extraction in a scoping review. ^50, 70– 73^ First, we conducted a narrative (literal) extraction of study characteristics (from the studies included in this review) including authors’ citation, country in which the study was conducted, participants’ characteristics, study sample size, study design, the name of the outcome measures used, intervention setting (for intervention studies only), and who delivered the intervention (for intervention studies only). Finally, a descriptive analytical process was used to search, extract, and summarise data regarding the clinical utility of each instrument across the domains previously described in the eligibility criteria (concept). Information regarding the clinical utility of postural outcome tools were obtained from the included studies and/or other studies reporting each of the domains of clinical utility for each postural outcome tool. Where further information was needed, this was retrieved from other publications including those of the original developers of the postural outcome tools. Characterisation/mapping of the outcome tools was performed by CNI-C and EUA. The illustration in Figure 2 summarises the data charting process.

### Synthesis of results

CNI-C and EUA independently extracted, coded, and interpreted information in relation to the clinical utility of the postural outcome tools using qualitative content analysis. These included construct validity (yes or no); predictive validity (yes or no); sensitivity to change (yes or no); ease of potential use in clinical settings (Yes-little or no training is required to use the tool or No-significant training required to use the tool, and Yes-no specialist equipment is required for the tool or No-specialist equipment is required for the tool); sensibility of the tool (Yes-significantly facilitates clinician-patient interaction and collaboration through requiring feedback from or active involvement of the patient in the assessment process or No-minimal or no facilitation of clinician-patient interaction and collaboration through little or no feedback from the patient/active involvement of the patient in the assessment process); the format of the tool [including the length of assessment (Easy-completed within 30 minutes or Hard-takes longer than 30 minutes to complete ^74^), wording of items (Simple-simple and easy to understand or Hard-complex terms were used and may be difficult to understand), response format or calibration of items (Easy-easy to complete with few response options or Difficult-difficult to complete with multiple response options)]; and the model of pain on which the postural outcome tool appears to be based (postural-structural-biomechanical model of pain based on the assumption that there are specific correct and wrong postures, or a biopsychosocial model of pain based on the assumption that postural aggravating factors are different in different individuals and may require individuals to identify what these are and actively modify exposure to identified aggravating postural factors ^24– 26^). Discrepancies in coding and synthesis were resolved by discussion of the entire review team including BCO and GNE.

## Results

### Selection of sources of evidence

**Figure 3.**
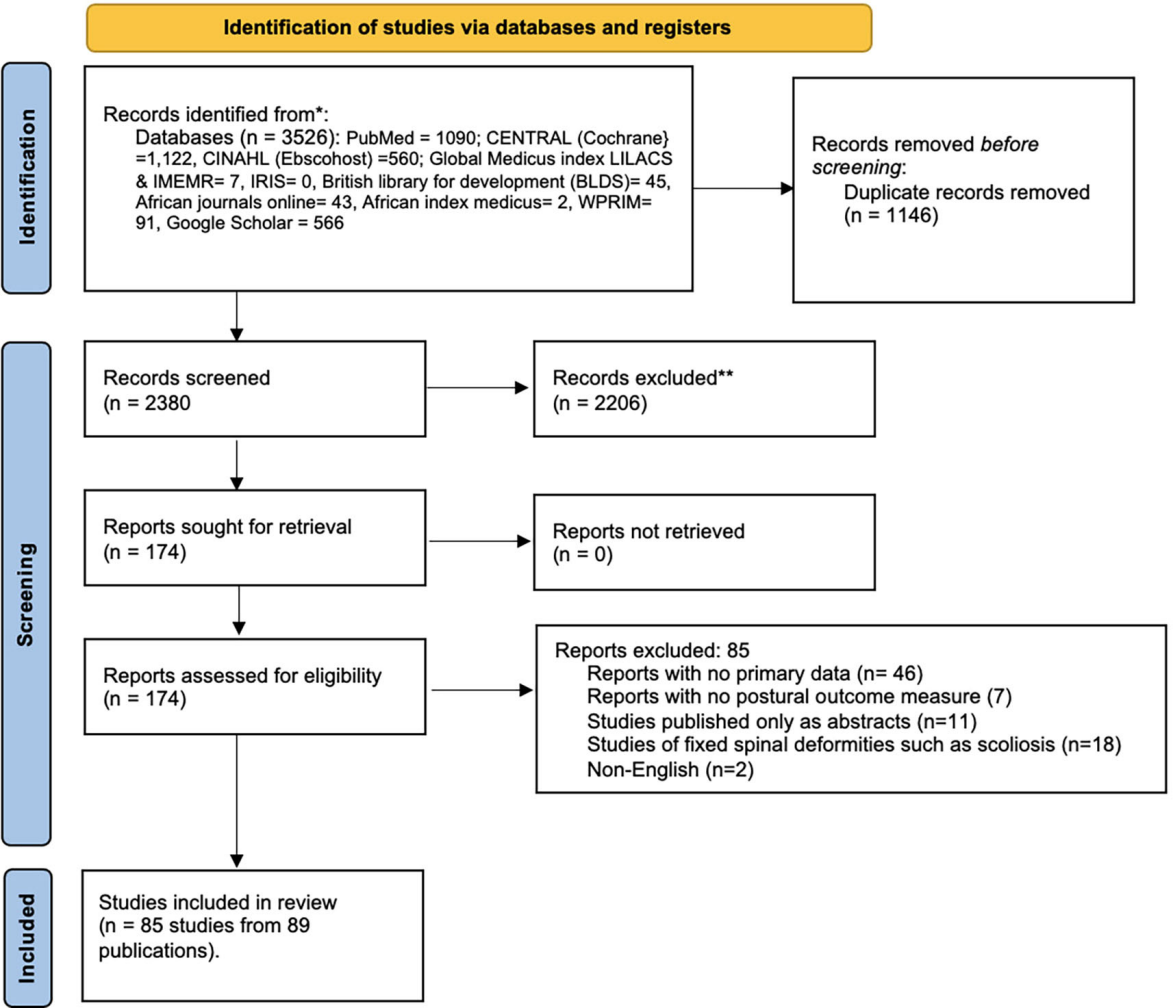
Flow diagram of the study selection process.

Figure 3 illustrates the process for the selection of studies included in this review. The initial search yielded 3,526 potential articles. 1,146 were removed during deduplication of records. The titles and abstracts of 2,380 papers were screened and 2,206 were excluded for not meeting the eligibility criteria. The full texts of 174 articles were assessed for eligibility with 85 full text articles excluded for failing to meet the eligibility criteria. A total of 85 studies (identified from 89 publications) included 50 cross-sectional studies, 9 pre-post-test studies; 11 RCTs, 7 prospective cohort studies, and 8 psychometric studies.

### Characteristics of sources of evidence and results of individual sources of evidence

Table 1 illustrates the characteristics of individual studies including the postural outcome tools that were utilised. The studies were published between 1995 and 2023 and were conducted in over 20 countries which included high, middle, lower-middle and low-income countries. Participants in the studies were aged 10 years and above with or without spinal pain. Twenty-eight specific postural outcome tools plus several self-developed (bespoke for individual studies) questionnaires, reports, observation, and demonstration were administered in the workplace, schools, computer laboratories, community centres, and hospitals or clinics in the included studies. There was a trend with more observational and psychometric studies using sophisticated computer-based or machine-based electronic devices, or complex time-consuming questionnaires and worksheets, and more intervention-based studies utilising mostly non-validated self-developed questionnaires specifically developed for each of the studies.

**Table 1.** Characteristics of the included studies.

Author (year)	Country	Age (years)	Sex	Educational level	Occupation	Sample size	Study design	Spinal condition	Outcome measures	Intervention setting	Intervention delivery
**Observational and psychometric studies**											
da Rosa et al., 2017 ^75^	Brazil	Year 1: 13.1 (SD 1.3) Year 2: 14 (SD 1.0) Year 3: 14.5 (SD 1.1) Year 4: 16 (SD 1.2)	M/F	5 ^th^ to 8 ^th^ grade	School children	42	Prospective cohort study	Back pain	DIPA by photogrammetry and BACKPEI	NA	NA
Igwesi- chidobe et al., 2017 ^18^	Nigeria	48.6 (SD 12.0)	M/F	Primary level	Mixed (unemployed, self-employed and paid work)	200	Cross sectional	Chronic low back pain	Occupational risk factor questionnaire	NA	NA
Mitchell et al, 2008 ^76^	Australia	22.5 (SD 4.5)	F	Nursing students	NS	170	Cross sectional	Low back pain	Electromagnetic measurement/electromagnetic tracking system in static postures and across a range of functional tasks.	NA	NA
Irwan et al., 2020 ^77^	Malaysia	66 (SD $3.8\;\left(66-83\right)$	M (98.9%)	Secondary school	Drivers	443	Cross sectional	Low back pain	Video footage of 15 minutes which was analysed with RULA + QEC Approach	NA	NA
Anita et al., 2014 ^78^	Malaysia	<25	M	Higher school certificate & diploma	Assembly line workers	232	Cross sectional	neck, upper back pain, low back pain	RULA	NA	NA
Romlah et al., 2020 ^79^	Indonesia	26-30	M/F	Nursing qualification	Emergency department nurses	40	Cross sectional	Neck pain and upper back pain.	REBA	NA	NA
Labbafinejad et al., 2016 ^80^	Iran	34 (SD 9.47)	M	NS	Employees of packaging units at pharmaceutical companies	396	Cross sectional	Lower back & neck pain	RULA	NA	NA
Chowdhury et al., 2012 ^81^	India	NS	NS	NS	Liquid Petroleum Gas workers	100	Cross sectional	Lower back pain	OWAS and a self-developed questionnaire	NA	NA
Ozdemir et al., 2021 ^82^	Turkey	16.67 (SD $1.1\left(15-19\right)$	M/F	9 ^th^ –12 ^th^ grade	High school students	2,743	Cross sectional	Back pain	BACKPEI	NA	NA
Ncube et al., 2019 ^83^	Zimbabwe	23.2 (SD 1.6 )	M/F	Undergoing University education	Undergraduate students	100	Cross sectional	Lower back pain, neck pain	RULA	NA	NA
Shah et al., 2016 ^84^	Pakistian	NS	M/F	NS	Garments manufacturing workers	80	Cross sectional	Neck pain, elbow pain, forearm pain, wrist/hand pain, thigh pain, ankle/feet pain	RULA & REBA	NA	NA
O’sullivian et al., 2006 ^85^	Australia	38.24 (SD 9.33)	M	NS	Industrial workers	45	Cross sectional	Low back pain and a control group with no pain	Self-developed questionnaire to assess commonly adopted postures, both at work and home	NA	NA
Inoue et al., 2020 ^86^	Japan	40 (SD 11.6)	M/F	NS	Manufacturing company workers	691	Cross sectional	Low back pain	Self-reports	NA	NA
Tan et al., 2020 ^87^	Malaysia	39 (SD 10.80)	M/F	NS	Restaurant Chef	126	Cross sectional	Low back pain, shoulder pain, ankle and foot pain	REBA	NA	NA
Dankaerts et al., 2006 ^88^	Australia	No LBP: 32.0 (SD 12.2), Flexion pattern: 35.7 (SD 11.2), Active extension pattern: 39.9 (SD 11.3)	M/F	NS	NS	57	Pre-post-test	Non-specific chronic low back pain	Electromagnetic measurement device	NA	NA
Khandan et al., 2018 ^89^	Iran	36-50	M/F	NS	Drug producers, workers in printing and publishing houses, dairy, and drinks producers	455	Cross sectional	Low back pain (most prevalent)	RULA, REBA & NERPA	NA	NA
Farahmand et al., 2020 ^90^	Iran	Prosthetist: Male 40.07 (SD 7.7);Female37.6 $\left(\mathrm{SD}\;7.4\right),\text{Orthotist}$ Male42.6 $\left(\mathrm{SD}\;4.4\right)$ Female37.64 SD3.8	M/F	NS	Prosthetists and Orthotists	40	Cross sectional	Neck pain, shoulder pain, elbow pain, wrist and hand pain, back pain, pelvic and thigh pain, knee pain, ankle and foot pain	REBA	NA	NA
Golchha et al., 2014 ^91^	India	36	M/F	University degree	Dentists	104	Cross-sectional	Spinal pain	RULA	NA	NA
Reza et al., 2021 ^92^	Malaysia	NS	NS	NS	Industry workers	45	Cross-sectional study	NS (spinal pain prevention?)	REBA	NA	NA
Burt et al., 1999 ^93^	USA	NS	NS	NS	Workers in a automotive manufacturing facility	NS (different jobs but not individuals were evaluated)	Cross-sectional	NS (spinal pain prevention?)	Observation of posture by two observers	NA	NA
Sarkar et al., 2016 ^94^	India	Group A: 27.5 (SD 3.7), Group B: 37.4 (SD 4.9), Group C: 45.6 (SD 6.7)	NS	NS	Manual material handling (MMH) workers in a market area	100	Cross- sectional	Low back pain, neck pain, knee pain, shoulder pain	OWAS	NA	NA
Filho et al., 2015 ^11^	Brazil	16.8	M/F	High school	High school students	1102	Cross-sectional	Low back pain, acute low back pain, chronic low back pain	Self-developed questionnaire with images, observation	NA	NA
Petrak et al., 2015 ^95^	Croatia	20-35	M	NS	NS	50	Cross-sectional	Nil pain – study aimed to develop a parametric garment pattern which can be adjusted according to the individual measurements of test subjects with different irregular body postures.	3D body scanner and innovative computer-based method for 3D body model analysis which enabled the assessment of body posture and very precise measurement of parameters for body posture assessment.	NA	NA
Widiyawati et al., 2020 ^96^	Indonesia	21 $\left(\mathrm{SD}\;4\right)\;$	NS	NS	Construction workers and managers of network infrastructure, a Telecommunication Service	33	Cross-sectional	Neck pain, upper back pain, and lower back pain.	RULA	NA	NA
Akodu et al., 2015 ^97^	Nigeria	43.45 (SD 7.55) (22-57)	M/F	NS	Office secretaries	150	Cross sectional	Neck pain, low back pain	RULA	NA	NA
Yung-hui & ko- chiou, 1995 ^98^	NS	24.8	F	Nursing qualification	Nurses	64	Cross-sectional	Low back pain	OWAS system & photogrammetry	NA	NA
Pan et al., 1999 ^99^	USA	NS	M/F	NS	Retail merchandise store workers	134	Cross-sectional	Back pain	PATH measurement method and observation	NA	NA
Van Oostrom et al., 2012 ^100^	The Netherlands	26-65	M/F	low (intermediate secondary education or less): 54.2% medium (intermediate vocational or higher secondary education): 26.6% High (higher vocational education or university): 19.2%	NS	5706	Prospective cohort	Chronic low back pain	Self-developed questionnaire	NA	NA
Perry et al., 2008 ^101^	Australia	13-17	M/F	NS	NS	22	Psychometric	No existing pain (spinal pain prevention) – study aimed to assess the reliability of photographic spinal posture assessment in adolescents	Photogrammetry	NA	NA
Mallare et al., 2017 ^102^	Philippines	16-24	M/F	NS	NS	42	Cross-sectional	No existing pain (spinal pain prevention)	Photogrammetry using a software that measures and assesses the sitting posture parameters	NA	NA
Fortin et al., 2018 ^103^	Canada	NS	NS	University education	Clinicians (Physical therapists and Sports therapists)	32	Prospective cohort	Nil pain (spinal pain prevention) – study aimed to determine the level of acceptance of the CPPAT and to document predictors as well as facilitators of and barriers to the acceptance of this tool by clinicians doing posture re-education	Photogrammetry: clinical photographic posture assessment tool (CPPAT). CPPAT is a software-based program with a graphical interface for the analysis of four to six photographs of a patient’s posture (front, back, left and right) acquired in standing using a standard procedure	NA	NA
Taylor, 2021 ^104^	USA	18-24	F	Undergraduate studies	Dental hygiene students	35	Cross-sectional	Nil pain (spinal pain prevention) – study aimed to to assess biomechanical and postural impacts of sitting and standing independently during dental hygiene practice	Photogrammetry and RULA. Two images per session, for a total of four images per participant, were taken to assess biomechanical demands of each posture using the Rapid Upper Limb Assessment (RULA) tool	NA	NA
Branson et al., 2002 ^105^	USA	NS	NS	NS	NS	NS	Psychometric	NS (spinal pain prevention)	PAI	NA	NA
Nam et al., 2013 ^106^	Korea	21.3 (SD 1.7)	M/F	Undergraduate studies	Undergraduate students	45	Psychometric	Neck pain	Photogrammetry	NA	NA
Stolinski et al., 2017 ^107^	Poland	Group 1: 7-10 (8.2; $\mathrm{SD}\;1.0);\text{Group}\;2:7-10;\left(8.4;\mathrm{SD}\;0.5\right)$	M/F	Primary	Primary school students	91	Psychometric	Nil pain (spinal pain prevention) – study aimed at presenting a structured method for the analysis of posture and its changes using a standardized digital photography technique	Photogrammetry	NA	NA
Brazil et al., 2022 ^108^	USA	NS	F	NS	NS	52	Cross-sectional	NS (spinal pain prevention)	RULA	NA	NA
Alvarez et al., 2021 ^109^	Ecuador	NS	M	NS	Craftsmen	15	Cross-sectional	NS (spinal pain prevention)	RULA and OWAS	NA	NA
Kee & Karwowski, 2007 ^110^	Korea	NS	NS	NS	Workers in manufactoring industries including iron, steel, electronics, automotive, chemical and service industries	NS (postures rather than the number of people were highlighted)	Cross-sectional study	NS (spinal pain prevention)	OWAS, RULA, REBA.	NA	NA
Chung et al., 2002 ^111^	Korea	22.6 (SD1.1 )	M	NS	NS	19	Pre-post-test/Quasi-Experimental with no intervention	Neck, and low back.	Computer based posture evaluation	NA	NA
Driel et al., 2013 ^112^	Canada	22 (18-28)	M	NS	Staff and student volunteers at a university	8	Cross-sectional study	No reported pain (spinal pain prevention)	Motion analysis system & inclinometer (simple data-logging inclinometer)	NA	NA
Freitag et al., 2007 ^113^	Germany	NS	NS	NS	Nurses	NS	Cross-sectional	NS (spinal pain prevention)	CUELA system	NA	NA
Kong et al., 2018 ^114^	Korea	NS	NS	NS	NS	NS	Cross-sectional	NS (spinal pain prevention)	ALLA, REBA, RULA, OWAS	NA	NA
Vidal-Conti et al., 2021 ^115^	Spain	NS	M/F	NS	Primary school teachers	85	Cross sectional	Low back pain	Self-developed LKQ	NA	NA
Kelly et al., 2009 ^116^	Ireland	NS	M/F	NS	Secondary school students	40	Cross-sectional	Musculoskeletal discomfort including spinal pain	RULA	NA	NA
Odole et al., 2020 ^117^	Nigeria	19.67 $\;\left(\mathrm{SD}\;2.1\right)$	M/F	Undergraduate studies	Undergraduate students	400	Cross sectional	Low back pain, neck pain, shoulder pain, wrist pain	Plumbline	NA	NA
Brink et al., 2015 ^118^	South Africa	16.3 $\;\left(\mathrm{SD}\;0.5\right)\;$	M/F	High school	Grade 10 high-school students	240	Prospective cohort	Neck pain	Photogrammetry-3D posture	NA	NA
Barbeito et al., 2021 ^119^	Spain	9-11	M/F	NS	NS	300	Cross sectional	Spinal pain (including cervical pain, dorsal and lumbar pain)	Self-developed questionnaire about postural hygiene	NA	NA
Sedrez et al., 2015 ^120^	Brazil	7-18; mean 12.9 (SD 2.3)	M/F	NS	High school students	59	Cross sectional	No existing pain (spinal pain prevention) – study investigated the association between behavioral risk factors, specifically postural habits, with the presence of structural changes in the spinal column of children and adolescents	BackPEI	NA	NA
Deokhoon et al., 2019 ^121^	Australia	33.9 [SD 11.8]	M/F	NS	Office workers	31	Psychometric	Nil neck pain	Motion sensor	NA	NA
Monfort-Pañego, 2020 ^122^	Spain	13 & 17 (14.47 (SD 1.26)	M/F	Secondary	NS	168	Psychometric	No existing pain (spinal pain prevention) – developed questionnaire used to assess back-health-related postural habits in the daily activities of adolescents	BEHALVES	Computer laboratory	NA
Kelly et al., 2022 ^123^	USA	NS	NS	NS	Surgeons	7	Psychometric	No existing pain – neck pain prevention is the focus.	Craniovertebral angle was measured using photogrammetry.	Hospital (surgical department)	NA
Lin et al., 2023 ^124^	China	NS	NS	NS	Surgeons	3	Cross-sectional (comparative)	No existing pain – prevention of pain in the neck, trunk, legs, upper arm, lower arm, and wrist.	Posture was measured with REBA	Hospital (surgical setting)	NA
Bahat et al., 2023 ^125^	Israel	Neck pain group 38.1 (SD 9.9) Control group 33.6 (SD 7.7)	M/F	NS	Staff and students of a university	43	Cross-sectional (comparative)	Two groups – neck pain group and the other group without neck pain.	Craniovertebral angle was measured using photogrammetry.	University	NA
Trkov et al., 2022 ^126^	USA	29.7 (SD 3.2)	M	NS	NS	6	Psychometric	No existing pain – back pain prevention targeted	Algorithm was used to measure posture plus spinal loading based on measurements from instrumented insoles and a single chest-mounted accelerometer.	NS	NA
Sriagustini, & Supriyani, 2022 ^127^	Indonesia	32 – 71	M/F	NS	Bamboo Craftsmen	6	Cross-sectional	No existing pain – spinal pain prevention targeted	RULA	Workstations	NA
Schwertner et al., 2022 ^128^	Brazil	15 – 18	M/F	High school students	High school students	679	Cross-sectional	Low back pain	Questionnaire of Body Awareness and Postural Habits of Young People (Q-BAPHYP).	School	NA
Sarraf & Varmazyar, 2022 ^129^	Iran	19 – 29	M/F	University students	University students	80	Cross-sectional	Neck pain	Photogrammetry was used to measure head and neck tilt angles, the gaze angle, and the change of the forward head posture.	University	NA
Cheragh et al., 2023 ^130^	Iran	39.95 (SD 5.30)	F	NS	Office employees	22	Cross-sectional	Chronic non-specific neck pain	Inclinometer	Workstations	NA
Fischer et al., 2022 ^131^	Brazil	Low back pain group 20.85 (SD 1.69) Control group without low back pain 20.05 (SD 2.54)	F	Undergraduate students	Undergraduate students	40	Cross-sectional (comparative)	Low back pain	Photogrammetry	University	NA
White et al., 2022 ^132^	USA	18 – 85	M/F	NS	NS	79	Cross-sectional (comparative)	Neck pain and those without neck pain	Forward head posture was measured with Cervical Range Of Motion (CROM) device	Community centre	NA
Hayati et al., 2022 ^133^	Iran	47 (SD 4.0)	M	NS	Manual workers – Leafy vegetable cultivation (LVC)	31	Cross-sectional	Low back pain and other musculoskeletal complaints	OWAS	Workstations	NA
Tomar et al., 2022 ^134^	India	Males 33.3 (SD 10.6) Females 27.1 (SD 5.3)	M/F	NS	Beauty salon workers	240	Cross-sectional	Neck pain, back pain, and pain in the shoulder, elbow, wrist/hand, legs and ankles/feet.	Self-developed survey of working postures	Workstations	NA
Pattath & Webb, 2022 ^135^	USA	21 – 55 and above	M/F	Graduate and undergraduate students	University students	338	Cross-sectional	Neck pain, lower back pain, upper back pain, shoulder pain, wrist pain,	Self-developed question on awkward or poor posture	University	NA
**Intervention studies**											
Wade, 2018 ^136^	USA	NS	M/F	NS	School teachers	NS	Pre-post-test/Mixed-methods secondary analyses of before and after effects of an educational programme	No existing pain (spinal pain prevention) – programme aimed at increasing postural awareness amongst schoolteachers	Self-developed questionnaire for assessing preliminary knowledge on posture and postural principles	School	Researchers
Cardon et al., 2007 ^137^	Belgium	9.7 (SD 0.7), range 8.1–12.0)	M/F	fourth- and fifth grade	NS	555	Pre-post-test	No existing pain (spinal pain prevention) – programme aimed at preventing back pain	Posture observation plus a self-developed questionnaire plus video and coding of movement sessions	School	Physical therapist
Llona et al., 2014 ^138^	Spain	Mean age:13.97 (SD 2.29)	M/F		School children	139	Prospective cohort	Back pain	Numerical pain scale and self-developed survey questionnaire	School	NS
Menor- Rodriguez et al., 2022 ^139^	Spain	6-12	M/F	primary	School children	479	Pre-post-test	No existing pain (spinal pain prevention) – programme aimed to improve postural hygiene in school children	Questionnaire containing questions from the program PERSEO [no explanation in English] and the ENKID study [no explanation in English], which were adapted with pictograms	School	NS
Candotti et al., 2011 ^140^	Brazil	Children: 10.5 (SD 0.8), Adolescence 13.2 (SD 1.0)	M/F	NS	School children	34	RCT	No existing pain (spinal pain prevention) – programme aimed to improve postural hygiene and knowledge about the spine in school children	Photogrammetry-Photography for static posture, recording of dynamic postures, and self-developed questionnaire about the theoretical knowledge of the spine	NS	NS
Foltran et al., 2011 ^141^	Brazil	9-16	M/F	4 ^th^ – 8 ^th^ grade	School children	392	Pre-post-test	No existing pain (spinal pain prevention) – programme aimed to improve schoolchildren's knowledge regarding back pain prevention	Self-developed questionnaire with eight out of ten questions based on identification of correct postures in illustrations	School	Physical therapist
Galmes- panades et al., 2022 ^142^	Spain	10-12	M/F	5 ^th^ & 6 ^th^ grade	School children	253	RCT	No existing pain (spinal pain prevention) – programme aimed to improve postural habits of school children	Self-developed questionnaires to investigate daily postural habits	Classroom/School	NS
Vidal et al., 2011 ^143^	Spain	10-12	M/F	5 ^th^ & 6 ^th^ grade	School children	145	RCT	No existing pain (spinal pain prevention) – programme aimed to improve postural habits of school children	Self-developed questionnaire that assessed correct use of sofa, stooping correctly, take care to sit correctly at home/school and frequent posture change on chair at home/school. A sum score was computed from the 6 items.	School	NS
Robbins et al., 2009 ^144^	United Kingdom	11-12	M/F	Secondary	School children	71	RCT	LBP and no pain in others – programme aimed to reduce the the reported severity of discomfort of musculoskeletal problems and the frequencies of musculoskeletal problems.	Self-developed questionnaire-A posture quiz using the electronic Classroom Performance Software system consisting of key- pads for each student to choose answers for a set of 10 multiple choice questions. This provided instant feedback on student understanding of the lesson content.	School	NS
Hill, 2015 ^145^	New Zealand	8-11	M/F	Primary	NS	469	Prospective cohort study	NS (spinal pain prevention?) – programme aimed to improve adherence to exercises designed to encourage movement of the lumbar spine through flexion, extension, and lateral flexion.	Self-reports and demonstration: demonstrated an understanding of good posture, remembered key points from previous sessions, demonstrated by repeating the exercises accurately and demonstrating good posture to the researcher.	School	NS
Ritter & de-souz, 2015 ^146^	Brazil	Mean:14 (SD 0.93)	M/F	NS	NS	32	Pre-post-test	No existing pain (spinal pain prevention) – programme aimed to to verify the short- and long-term effectiveness of the Elementary School Postural Program in the performance, generalization, and perception of daily school activities	Video recording and self-administered questionnaire. Self-developed Tool for Knowing How Students Perceive Posture to determine the level of awareness of participants for their own posture while performing specific school activities (the act of sitting; the act of sitting to write at a desk; the act of carrying school possessions; and the act of picking up light and heavy objects from the floor)	School	NS
Mendez et al., 2001 ^147^	NS	9	NS	3 ^rd^ grade	NS	106	Pre-post-test	No existing pain (spinal pain prevention) – the programme aimed to improve the level of knowledge and motor skills and thereby avert the development of painful symptoms after completion of a postural hygiene program	Self-developed questionnaire of 72 items referring to the anatomy of the spine, spine biomechanics, respiratory system, and spine overload.	NS	NS
Alexandre et al., 2002 ^148^	New York	46.5 (SD 12.2)	M/F	NS	NS	120	Prospective cohort	Back pain	Self-developed diary sheet	NS	Physiotherapist
Ribeiro et al., 2020 ^149^	New Zealand	45.3 (SD 13.2)	F	NS	Health care workers	130	RCT	No existing pain (spinal pain prevention) – the programme aimed to reduce the number of times forward bending posture is adopted at work.	Postural monitor and feedback device	Workplace	NS
Jaromi et al., 2012 ^150^	Hungary	24-57	M/F	NS	Nurses	124	RCT	Chronic low back pain	Photogrammetry	University clinic	NS
Albaladejo et al., 2010 ^151^	Spain	Control: 52.5 (45.0; 61.7) Education intervention: 51.0 (42.0, 58.0); Education and Physiotherapy intervention 51.0 (42.0; 59.7).	NS	NS	NS	348	RCT	Low back pain	Postural hygiene booklet	School	Physiotherapist
Cervera- Espert et al., 2018 ^152^	Spain	22.3 (SD 3.2)	M/F	undergraduate& postgraduate	NS	336	Cross sectional	No existing pain (spinal pain prevention) – the programme aimed to evaluate knowledge in relation to ergonomics about BHOP (balanced human operator position) concept and its application to routine clinical practice amongst undergraduate and postgraduate dental students	Self-developed questionnaire about basic knowledge of ergonomics	School	NS
Moustafa et al., 2022 ^153^	Egypt	Intervention group 25.1 (SD 3.0) Control group 24.0 (SD 4.2)	M/F	NS	NS	80	RCT	Chronic non-specific neck pain	4D formetric device was used to measure thoracic kyphosis angle	University clinic	Physiotherapist
Youssef et al., 2022 ^154^	China	Intervention group 27.4 (SD 5.5) 27.2 (SD 4.7)	M/F	NS	Mixed (university students, desk office workers, house wife)	24	RCT	Nonspecific neck pain	3D postural assessment with Global Posture System	Hospital (Rehabilitation department)	NS
Türkmen et al., 2023 ^155^	Turkey	Intervention group 34.3 (SD 4.3) Control group 35.8 (SD 2.8)	M/F	NS	NS	16	RCT	Neck pain with suboccipital headache	PostureScreen Mobile application	University clinic	Physiotherapist
Mohamed et al., 2022 ^156^	Germany	Intervention group 48.69 (SD 4.18) Control group 48.52 (SD 5.70)	M/F	NS	NS	70	RCT	Neck pain with cervical radiculopathy	Craniovertebral angle was measured using photogrammetry.	University clinic	Physiotherapist
Yong et al., 2023 ^157^	South Korea	Intervention group 41.4 (SD 5.19) Control group 43.5 (SD 5.22)	M/F	NS	NS	32	Pre-post-test	Chronic non-specific neck pain	Photogrammetry	NS	Physiotherapist

M: Male; F: Female; NS: Not Specified; USA: United States of America; SD: standard deviation; NA: Not applicable; LBP: low back pain; BackPEI: Back Pain and Body Posture Evaluation Instrument questionnaire; DIPA= Digital Imaged-based Postural Assessment; PPAM: Posture Analysis Method; ORFQ: Occupational risk factor questionnaire; BEHALVES, Back-hEalthrelated postural Habits in dAiLy actiVitiES; CUELA system: computer based measurement and long term analysis of stress upon musculoskeletal system; RULA: Rapid upper limb assessment; REBA: Rapid entire body assessment; OWAS: Ovako working posture analysis system; NERPA: novel ergonomic postural assessment method; CPPAT- Clinical photographic posture assessment tool; mRULA: modified rapid upper limb assessment; UC- checklist: use Computer; QEC: Quick Exposure Check; PATH-method: posture, activity tools and handling measurement method; RCT: Randomized Controlled Trial; intervention; CG= control group; PT= physiotherapy; UK: united Kingdom; US: United State;Non- RCT= non randomized control Trial; NS= not stated; STPM: Higher school certificate. System); ALLA= Agricultural lower limb assessment; PMF- device= Postural monitor and feedback device. PEP= postural Education Program; ICC: interclass coefficient correlate. PAI: Posture assessment instrument. LKQ: Low Back Pain Knowledge Questionnaire.

### Clinical utility of postural outcome tools identified from studies reporting the domains of clinical utility

**Figure 4.**
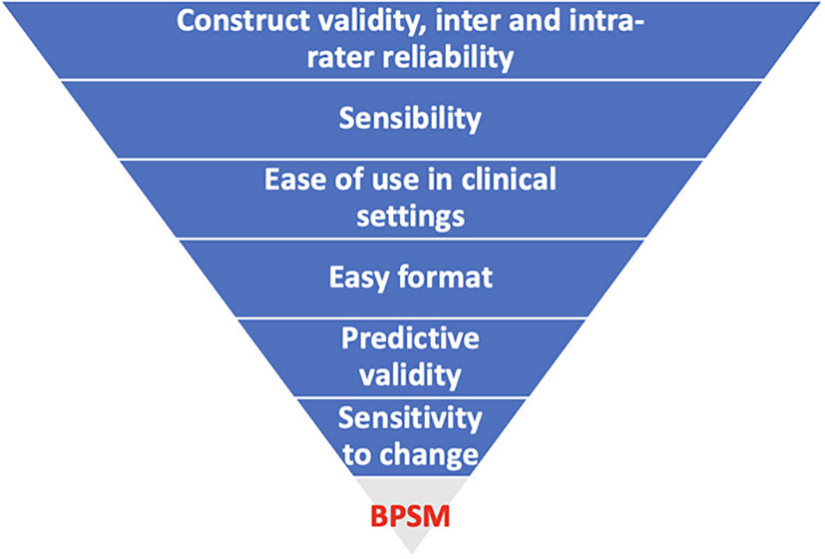
Clinical utility domains achieved.

**Figure 5.**
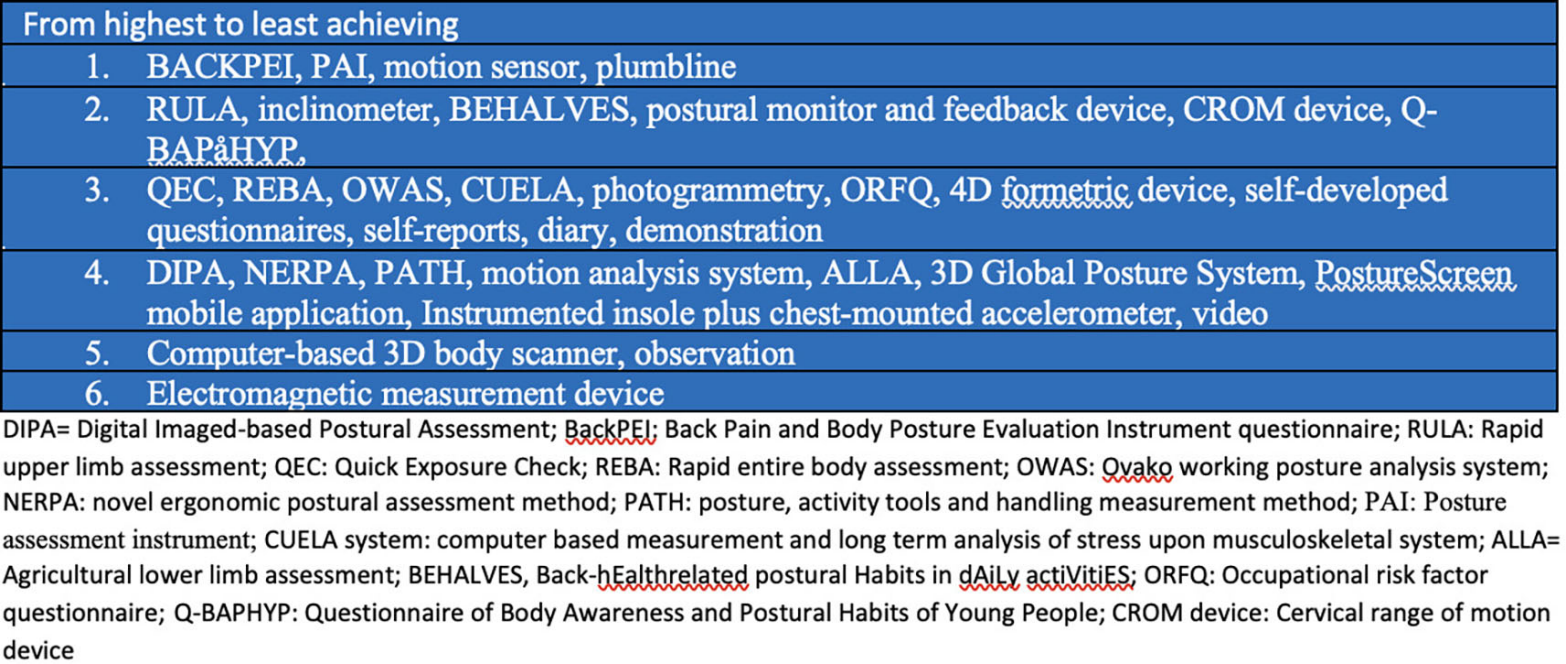
Achievement of overall clinical utility by the included postural outcome tools.

Table 2 highlights the clinical utility of the postural outcome tools that have been used in the studies. Notably, none of the postural outcome measures scored positively on all the domains of clinical utility. The domain of clinical utility in a decreasing order of achievement was construct validity and inter and intra-rater reliability (24 tools each), followed by sensibility (16 tools), ease of use in clinical settings (13 tools), easy format (13 tools), predictive validity (5 tools), sensitivity to change (2 tools), and underpinned by a biopsychosocial model of pain (0 tool). The highest achieving tools in relation to clinical utility are BACKPEI (5 domains), PAI (5 domains), motion sensor (5 domains), plumbline (5 domains); followed by RULA (4 domains), inclinometer (4 domains), BEHALVES (4 domains), postural monitor and feedback device (4 domains), CROM device (4 domains), Q-BAPHYP (4 domains), which were followed by QEC (3 domains), REBA (3 domains), OWAS (3 domains), CUELA (3 domains), photogrammetry (3 domains), ORFQ (3 domains), 4D formetric device (3 domains), self-developed questionnaires (3 domains), self-reports (3 domains), diary (3 domains), demonstration (3 domains), and after these were DIPA (2 domains), NERPA (2 domains), PATH (2 domains), motion analysis system (2 domains), ALLA (2 domains), 3D Global Posture System (2 domains), PostureScreen mobile application (2 domains), Instrumented insole plus chest-mounted accelerometer (2 domains), video (2 domains), and finally there were computer-based 3D body scanner (1 domain), observation (1 domain) and electromagnetic measurement device (0 domain). Notably, all the postural outcome tools were underpinned by a postural-structural-biomechanical model of pain based on the assumption that there are specific correct and wrong postures. None of the postural outcome measures was based on a biopsychosocial model of pain with an underlying assumption that postural aggravating factors may be different in different individuals and may require individuals to identify their own aggravating postures and possible interacting psychosocial and biomedical factors, and actively modify exposure to the identified aggravating postural factors. Figures 4 summarise the relative achievement of the domains of clinical utility by the included postural outcome tools. Figure 5 depicts the performance of the included postural outcome tools in achieving overall clinical utility.

**Table 2.** Clinical utility of the postural outcome tools.

Postural outcome tools	Brief description	Construct validity	Predictive validity	Intra-and/or inter-rater reliability	Sensitivity to change	Ease of potential use in clinical settings	Sensibility	Format	Model of pain on which the tool appears to be based
DIPA software	DIPA is a method that uses photogrammetry in combination with computer techniques to allow the assessment of different body segments in the sagittal and frontal planes hence providing clinical information of an individual’s posture. It involves assessing posture using digital images from different angles which are then analysed to determine an individual’s posture. ^158^	Yes ^159^	No: Evidence not found.	Yes ^159^	No: Evidence not found.	No: Significant training (3-5hours for fitness professionals) required to use the tool. The tool involves specialist equipment including image based computer apps, reflective markers for anatomical reference points and cameras. ^75, 103, 107^	No: Minimal or no facilitation of clinician-patient interaction and collaboration through little or no feedback from the patient/active involvement of the patient in the assessment process. ^75, 158, 159^	Hard: Takes longer than 30 minutes to complete. Requires computer software application and interpretation. ^75, 158, 159^	Postural-structural-biomechanical model of pain: Based on the assumption that there are specific correct and wrong postures. ^75, 158, 159^
BACKPEI	BACKPEI is a questionnaire that measures the prevalence of back pain and its associated risk factors which include socio-demographic, economic, genetic, behavioural, and postural risk factors. It was initially developed for use amongst school-age children ^160^ but expanded versions have been developed including neck pain amongst children and adolescents ^161^ and adults. ^162^ Postural risk factors assessed include posture in relation to sleeping, sitting in a chair to write, sitting in a chair to talk, using a computer and lifting an object from the ground. ^160^ The original and adapted versions of the questionnaire contain 20-30 items. ^160– 162^	Yes ^163, 164^	No: Evidence not found.	Yes ^160– 162^	No: Evidence not found.	Yes: Little or no training is required to use the tool. No specialist equipment is required for the tool. ^160– 162^	Yes: Significantly facilitates clinician-patient interaction and collaboration through requiring feedback from or active involvement of the patient in the assessment process. ^160– 164^	Easy: Can be completed within 30 minutes. The wording of the items are simple and easy to understand. The response format or calibration of items is easy to complete with few response option. ^160– 164^	Postural-structural-biomechanical model of pain: Based on the assumption that there are specific correct and wrong postures. ^160– 164^
RULA	The RULA method is a survey method of assessment designed for use in ergonomic evaluation of workplaces. It assesses biomechanical and postural loading on the whole body. Using the RULA worksheet, the evaluator assigns a score for each of the body regions: upper arm, lower arm, wrist, neck, trunk, and legs. Compilation of risk factors is completed after collating the data for each region and generating a single score which represents the overall level of musculoskeletal risk. The assessment involves first interviewing the worker, observing the worker’s movements and postures during work cycles, selection of the postures to be evaluated based on the most difficult postures and work tasks based on interview and observation, the posture sustained for the longest time, and the posture where the highest force loads occur. ^127, 165, 166^	Yes ^167– 169^	Yes ^170^	Yes ^167– 169^	No: Evidence not found.	No: Training is required to use the tool. Assessment requires going to the workplace and observing the worker’s movements and postures during several work cycles with or without photographs/videos taken. However, no specialist equipment is required for the tool. ^165– 169^	Yes: Aspects of the assessment facilitates clinician-patient interaction and collaboration through requiring feedback from or active involvement of the patient. ^165– 169^	Hard: Takes longer than 30 minutes to complete and requires observation in the workplace with or without analyses of photographs in the workplace. The wording of some items is complex. The response format or calibration of some items is complex. ^127, 165– 169^	Postural-structural-biomechanical model of pain: Based on the assumption that there are specific correct and wrong postures ^127, 165– 169^
QEC	QEC is a questionnaire designed for assessing changes in exposure to musculoskeletal risk factors of the back, shoulders and arms, hands and wrists, and neck before and after an ergonomic intervention. It involves the practitioner (the observer) conducting the assessment, and the worker who has a direct experience of the task. QEC is designed to indicate change in exposure scores following an ergonomic intervention. QEC has two sections – an observer’s assessment section of the questionnaire that involves the practitioner describing the posture of the back, shoulder/arm, wrist/hand, and neck during specific work tasks, and a worker’s assessment section which entails workers completing questionnaire items regarding specific work activities involving weightlifting and other biomechanical exposures. QEC involves the following steps: deciding on the task to be assessed, conducting the assessment, scoring the assessment, interpreting the scores and prioritising, and reassessing change following interventions. ^171– 174^	Yes ^171– 174^	No: Evidence not found.	Yes ^171– 174^	No: Evidence not found.	No: Training is required to use the tool. Assessment requires going to the workplace and observing the worker’s movements and postures during several work cycles with or without photographs/videos taken. However, no specialist equipment is required for the tool.	Yes: Aspects of the assessment facilitates clinician-patient interaction and collaboration through requiring feedback from or active involvement of the patient. ^171– 174^	Hard: Although it takes shorter than 30 minutes to complete, with simple wording of items and response options, QEC requires observation in the workplace with or without analyses of photographs in the workplace. ^171– 174^	Postural-structural-biomechanical model of pain: Based on the assumption that there are specific correct and wrong postures. ^171– 174^
REBA	The REBA is a single page worksheet used to evaluate required or selected body posture, forceful exertions, type of movement or action, repetition, and coupling (human-load interface). It provides a postural analysis system that is sensitive to musculoskeletal risks in a variety of tasks and requires an assessment of right and left sides of the body, although it might be possible to determine which side of the body has the greatest exposure to musculoskeletal risk factors. It involves the following steps: first interviewing the worker to understand work tasks, then observing the worker’s movements and postures during several work cycles, finally the posture to be evaluated is then selected based on i) the most difficult postures and work tasks based on a combination of the worker interview and initial observation ii) the posture sustained for the longest period of time and iii) the posture where the highest force loads occur. ^124, 175– 177^	Yes ^89, 178^:	No: Evidence not found.	Yes ^179^	No: Evidence not found.	No: Training and practice are required to use the tool. Assessment requires going to the workplace and observing the worker’s movements and postures during several work cycles with or without photographs/videos taken. However, no specialist equipment is required for the tool. ^180^	Yes: Aspects of the assessment facilitates clinician-patient interaction and collaboration through requiring feedback from or active involvement of the patient. ^175– 177^	Hard: Takes longer than 30 minutes to complete and requires observation in the workplace with or without analyses of photographs in the workplace. The wording of the items is complex. The response format or calibration of items is complex. ^175– 177, 179, 180^	Postural-structural-biomechanical model of pain: Based on the assumption that there are specific correct and wrong postures. ^124, 175– 177, 179, 180^
OWAS	OWAS is an observational method of postural analysis that relies on the observation of a worker’s postures during tasks at regular intervals. OWAS identifies the most common work postures for the back (4 postures), arms (3 postures), legs (7 postures), and the weight of the load handled (3 categories). Whole body posture is described by these body parts with a four digit-code. Using worksheets, the method classifies the postures into 252 possible combinations based on the worker’s back, arms, and legs postures and the load being handled. Each observed posture is classified by ascribing it to a posture code. From the code of each posture, a risk or discomfort assessment is developed by assigning it a risk category. The method determines the risk category of each posture individually after coding the postures. The risk or discomfort is assessed for each body part (back, arms, and legs) together via considering all adopted postures. This is achieved through assigning each body part a risk category based on the relative frequency of various postures adopted in different observed postures. Finally, by analysing the risk categories for each observed posture and different body parts collectively, the most critical postures and positions are identified which then informs the required corrective actions or interventions to enhance the workstation. The application of the OWAS system involves observing the worker performing their task during which they carry out different activities which are then divided into distinct phases. The division into phases is required when worker’s activities change at different times. This assessment can be simple for homogenous tasks with consistent activities or multi-phase for non-homogenous work tasks with different activities or phases. Each phase is then evaluated separately. Observation time is usually standardised. Shorter observation period is required for jobs with brief, repetitive work cycles, whereas longer observation period is required for jobs with diverse tasks and undefined cycles. Observation period usually range between 20 and 40 minutes. The application of the OWAS involves the following steps: determine whether the task will be divided into several phases – simple or multi-phase evaluation; establish the total observation time for the task depending on the number and frequency of adopted postures; determine the observation or sampling frequency; observation and posture recording using photographs and videos taken from adequate viewpoints; coding of the observed postures; calculating the risk category for each posture; calculate the percentage of repetitions or relative frequency of each position for each body part; calculate the risk category for each body part based on the relative frequency; based on the results obtained, determine the necessary corrective actions; re-evaluate the task using the OWAS method to determine the effectiveness of improvements if changes were introduced ^133, 181, 182^	Yes ^178, 183, 184^:	Yes ^178, 185^:	Yes ^186– 188^:	No: Evidence not found.	No: Training and practice may be required to use the tool. Assessment requires going to the workplace and observing the worker’s movements and postures during several work cycles with or without photographs/videos taken. However, no specialist equipment is required for the tool ^133, 178, 181– 188^	No: Minimal or no facilitation of clinician-patient interaction and collaboration through little or no feedback from the patient/active involvement of the patient in the assessment process ^133, 178, 181– 188^	Hard: Takes longer than 30 minutes to complete and requires observation in the workplace with or without analyses of photographs in the workplace. The wording of the items is complex. The response format or calibration of items is complex ^133, 178, 181– 188^	Postural-structural-biomechanical model of pain: Based on the assumption that there are specific correct and wrong postures ^133, 178, 181– 188^
NERPA	NERPA is a postural assessment method that is adapted from the RULA and was developed using a 3-dimensional computer-aided design tool commonly used in the aeronautic and automotive industries. It was designed to detect and evaluate the potential risks experienced by workers due to musculoskeletal problems resulting from poor ergonomic design. Although the assessment structure used in the RULA method was retained in the NERPA, some joint ranges used in the RULA were modified for use in the NERPA method. Consequently, NERPA can detect more postures with ergonomic risk and is more sensitive to detecting postures with low ergonomic risk, although the RULA method might be better in predicting the risk of musculoskeletal disorders. ^170, 189– 191^	Yes ^170, 191^	No: Evidence not found.	No: Evidence not found.	No: Evidence not found.	No: Training is required to use the tool. Assessment requires going to the workplace and observing the worker’s movements and postures during several work cycles with or without photographs/videos taken. Computer software is needed to administer the assessment. ^170, 189– 191^	Yes: Aspects of the assessment facilitates clinician-patient interaction and collaboration through requiring feedback from or active involvement of the patient. ^170, 189– 191^	Hard: Takes longer than 30 minutes to complete and requires observation in the workplace with or without analyses of photographs in the workplace. Requires computer software application and interpretation. The wording of some items is complex. The response format or calibration of some items is complex. ^170, 189– 191^	Postural-structural-biomechanical model of pain: Based on the assumption that there are specific correct and wrong postures. ^170, 189– 191^
PATH	PATH is an observation-based work sampling-based ergonomic assessment approach developed to characterise the ergonomic risk factors that may cause musculoskeletal disorders at construction and other non-repetitive work. The ergonomic risk factors are in relation to the back, neck, lower limbs, and shoulders. PATH uses posture codes based on the OWAS method with additional codes included for describing worker activity, tool use, loads handled and grasp type. For heavy highway construction, observations are stratified by construction stage and operation, using a taxonomy developed specifically for this purpose. Observers can code the physical characteristics of the job reliably after 30 hours of training. The method involves data coding on a data collection sheet for each observation, posture, activity, and handling. The coded data output is customised for each combination of trade and operational activity. To carry out data collection, each observer selects a number of workers (preferably a crew) performing the same operation. The crew is usually followed for 3 or 4 hours during each sampling period (from beginning of shift to break or from break to end of shift). Observations are made at fixed intervals of usually 45 or 60 s. Forty-five s is the minimum interval used that maintains reliability. The specific worker for each observation is randomly determined from those selected for analysis at the start of the day. The task in which the specified worker is engaged is recorded, along with PATH data, at each observation. This random sampling of workers and tasks allows simultaneous observation of the proportion of time that workers in a specific trade perform each task during a specific operation, as well as the frequency of exposures in each task. ^192– 195^	Yes ^196^	No: Evidence not found.	Yes ^197^	No: Evidence not found.	No: 30 hours of training is required to use the tool. Assessment requires going to the workplace and observing the worker’s movements and postures during several work cycles with or without photographs/videos taken. Involves the use of a complex taxonomy. ^194^	No: Minimal or no facilitation of clinician-patient interaction and collaboration through little or no feedback from the patient/active involvement of the patient in the assessment process. ^192– 195^	Hard: Takes longer than 30 minutes to complete and requires observation in the workplace with or without analyses of photographs in the workplace. The wording of the items and the taxonomy is complex. The response format or calibration of items is complex. ^192– 197^	Postural-structural-biomechanical model of pain: Based on the assumption that there are specific correct and wrong postures. ^192– 197^
PAI	PAI is an instrument used in evaluating operator performance in the dental and dental hygiene education setting to determine the effects of operator posture and musculoskeletal disorders. The tool measures neutral and non-neutral operator positions in dental practice which might have associations with musculoskeletal discomfort. It semi-quantitatively assesses dental operator posture. ^105^	Yes ^198– 200^	No: Evidence not found.	Yes ^198– 200^	No: Evidence not found.	Yes: Little or no training is required to use the tool. No specialist equipment is required for the tool. ^105, 198– 200^	Yes: the assessment facilitates clinician-patient interaction and collaboration through requiring feedback from or active involvement of the patient. ^105, 198– 200^	Easy: Can be completed within 30 minutes. The wording of the items are simple and easy to understand. The response format or calibration of items is easy to complete with few response options. ^105, 198– 200^	Postural-structural-biomechanical model of pain: Based on the assumption that there are specific correct and wrong postures. ^105, 198– 200^
Motion analysis system	This is a 3D computer movement analysis system that conducts biomechanical analysis eliminating human error. The system records key biometric information about patients including gender, handedness, age, weight, and height which is used to build a real-time, dynamic 3D model of the patient’s body with enhanced accuracy. The result of the analysis is used to determine treatment is needed to correct asymmetries or not. The measurement involves attaching four sensors to the wrists and ankles such that when the patient stands in front of a camera, these are detected, and the system becomes calibrated and ready to use. The practitioner selects a test which the patient performs one to three times. The patient can view their balance lines, joint angles and joint loading in real time which are displayed on a monitor. Possible results displayed can include right-left imbalance, range of motion, knee valgus, peak joint loads etc. the test can be repeated for different postures and movements. A comprehensive report is generated with the click of a button and can include images of each test with results and comments. ^112^	Yes ^201– 204^	No: Evidence not found.	Yes ^201, 203^	No: Evidence not found.	No: Training is required to use the system. The tool involves specialist equipment including image based computer apps, sensors and reflective markers for anatomical reference points and cameras. ^112, 201– 204^	No: Minimal or no facilitation of clinician-patient interaction and collaboration through little or no feedback from the patient/active involvement of the patient in the assessment process. ^112, 201– 204^	Hard: Might take longer than 30 minutes to complete if analysing multiple postures and movements. Requires computer software application and sensors.	Postural-structural-biomechanical model of pain: Based on the assumption that there are specific correct and wrong postures. ^112, 201– 204^
Motion sensor	A motion sensor is an electronic device designed to detect and measure movement. Motion sensors are based on wearable technology that can track whole body postures for ergonomic assessments. They are usually small and lightweight with the ability to monitor postures over a long period of time. Motion sensors are usually embedded systems with three major components including a sensor unit, an embedded computer, and a hardware which is the mechanical component. These three parts usually vary size and can be customised to perform very specific functions. There are two types of motion sensors which include the active motion sensors and the passive motion sensors. Active motion sensors have a transmitter and a receiver and detects motion which is reflected into the receiver. A passive motion sensor does not have a transmitter, does not measure constant reflection, and detects motion based on a perceived increase of radiation in the sensor’s environment which is sent as electric data to the embedded computer and hardware component. They have been used in the diagnosis and treatment of musculoskeletal disorders. ^26, 205– 208^	Yes ^26, 207, 209^	Yes ^209^	Yes ^121^	No: Evidence not found.	No: Little or no training is required to use the tool. However, specialist equipment is required including the embedded systems with three major components including a sensor unit, an embedded computer, and a hardware which is the mechanical component. The cost of providing equipment for each patient might be prohibitive. ^26, 205– 209^	Yes: There is feedback from the patient/active involvement of the patient in the assessment process as the patient adjusts posture in relation to the feedback from the motion sensor. ^26, 205– 209^	Easy: Can be completed within 30 minutes and it is easy to interpret. ^26, 205– 209^	Postural-structural-biomechanical model of pain: Based on the assumption that there are specific correct and wrong postures. ^26, 205– 209^
Inclinometer	Inclinometer, also known as a tilt sensor, is a device used to measure the angles of slope, elevation, or depression of an object relative to the line of gravity. It can be used to assess and measure the motion of the spine. It assesses the anteroposterior curvature of the spine and can be used alone or in conjunction with a second tool for neck and back measurements. They can be used for diagnosis and for tracking the effects of treatments and rehabilitation. ^130, 210– 216^	Yes ^210– 213, 215, 216^	No: Evidence not found.	Yes ^211– 216^	No: Evidence not found.	Yes: Little or no training is required to use the tool. Some inclinometers are very cheap, commonly available, and can be administered by a variety of people in different professions. ^130, 210– 216^	No: Minimal or no facilitation of clinician-patient interaction and collaboration through little or no feedback from the patient/active involvement of the patient in the assessment process. ^130, 210– 216^	Easy: Can be completed within 30 minutes. The results are simple and easy to understand. The calibration is easy to interpret. ^130, 210– 216^	Postural-structural-biomechanical model of pain: Based on the assumption that there are specific correct and wrong postures. ^130, 210– 216^
CUELA	CUELA was designed for the measurement of musculoskeletal loads in the workplace such as manual load handling, awkward body posture or repetitive movements. It is an inertial sensor-based computer-based measurement system that has been adapted for long-term analysis of musculoskeletal workloads including joint range of motion in occupational settings. The system performs ambulatory assessment of physical workloads in occupational settings and facilitates the continuous recording and analysis of physical workloads at the workplace. It does this through recording and analysing posture and motion data whilst individuals are in their workplaces. It was designed for ergonomic field analysis by performing whole-shift recordings and then completing analysis of work-related postural and mechanical loads. CUELA records body and joint movements with the aid of motion sensors that are attached to the working person’s clothing. The measured data are digitalised in a data storage device and stored on flashcards. The data storage device has an online recording mode with which data can be sent via Bluetooth to a computer and visualised in real time. ^113, 217, 218^	Yes ^219, 220^	No: Evidence not found.	Yes ^219^	Yes ^221^	No: Significant training required to use the tool. The tool involves specialist equipment including sensors and computers. ^113, 217– 221^	No: Minimal or no facilitation of clinician-patient interaction and collaboration through little or no feedback from the patient/active involvement of the patient in the assessment process. ^113, 217– 221^	Hard: Takes longer than 30 minutes to complete. Requires computer software application and interpretation. ^113, 217– 221^	Postural-structural-biomechanical model of pain: Based on the assumption that there are specific correct and wrong postures. ^113, 217– 221^
ALLA	ALLA is an ergonomic evaluation tool used to assess lower limb postures associated with farming tasks Using the ALLA worksheet, the evaluator assigns different scores for each posture of the lower limb as well as the duration for which that posture is maintained. ALLA involves observing the worker’s movements and postures during work cycles. ^114, 222^	Yes ^114^	No: Evidence not found.	No: Evidence not found.	No: Evidence not found.	No: Training is required to use the tool. Assessment requires going to the workplace and observing the worker’s movements and postures during several work cycles with or without photographs/videos taken. However, no specialist equipment is required for the tool. ^114, 222^	Yes: Aspects of the assessment facilitates clinician-patient interaction and collaboration through requiring feedback from or active involvement of the patient. ^114, 222^	Hard: Takes longer than 30 minutes to complete and requires observation in the workplace with or without analyses of photographs in the workplace. The wording of some items is complex. The response format or calibration of some items is complex. ^114, 222^	Postural-structural-biomechanical model of pain: Based on the assumption that there are specific correct and wrong postures. ^114, 222^
Plumbline	Plumbline is a method of evaluating posture that involves using a vertical plumb line over the centre of the body from the top of the head to the floor to show alignment of specific points of the body through which the line of gravity is expected to pass through in an ideal posture. The plumbline is used to assess the midline of the body to detect asymmetrical and rotation faults. In an ideal situation, the plumbline should divide the body into equal and symmetrical halves. Using a posture examination checklist, a string suspended overhead with a small weight, or plumb bob, is attached at the end near the floor. The patient is positioned so that the body bisected by the plumb line. Anterior, lateral and posterior views are obtained. In the anterior view, the checklist is used to detect any deviations from normal in relation to the right and left halves of the body. In the lateral view, the checklist is used to identify any deviations from normal in relation to views obtained from both the left and right sides of the body. In the posterior view, right and left halves of the body are considered from the posterior part of the view with considerations to arch positions, knee fossa alignment, scoliosis, scapula height etc. ^223^	Yes ^224^	Yes ^225^	Yes ^224, 226^	No: Evidence not found.	Yes: Little or no training is required to use the tool. Plumbline is very cheap, commonly available, and can be administered by a variety of people in different professions. ^223– 226^	No: Minimal or no facilitation of clinician-patient interaction and collaboration through little or no active involvement of the patient in the assessment process as this is a passive assessment process. ^223– 226^	Easy: Can be completed within 30 minutes. The results are simple and easy to understand. The calibration is easy to interpret. No specialist equipment is required. ^223– 226^	Postural-structural-biomechanical model of pain: Based on the assumption that there are specific correct and wrong postures. ^223– 226^
BEHALVES	BEHALVES is a questionnaire that assesses the postural habits in the daily activities of adolescents. The items are grouped into five categories: standing posture (items 1-4), sitting posture (items 5–13), use of backpacks (items 14–20), mobilizing heavy weights (items 21–26) and lying posture (items 27–31). The items in the questionnaire are scored with: 1 = Never, 2 = Hardly ever, 3 = Almost always and 4 = Always. Questions 4, 6, 7, 8, 9, 27, 30 are scored inversely. ^122^	No: Evidence not found.	No: Evidence not found.	Yes. ^122^	No: Evidence not found.	Yes: Little or no training is required to use the tool. No specialist equipment is required for the tool. ^122^	Yes: Significantly facilitates clinician-patient interaction and collaboration through requiring feedback from or active involvement of the patient in the assessment process. ^122^	Easy: Can be completed within 30 minutes. The items are simple and easy to understand. The calibration is easy to interpret. No specialist equipment is required. ^122^	Postural-structural-biomechanical model of pain: Based on the assumption that there are specific correct and wrong postures. ^122^
Photogrammetry	Photogrammetry is the art, science, and technology of information on physical objects and environment through the processes of recording, measuring, and interpreting photographic images and patterns of electromagnetic radiant energy and other sources. It is a non-invasive technique for postural evaluation that provides measurements of body angles or distances which allow for quantitative posture assessment with or without the use of external markers. Photogrammetry quantifies postural assessment by measuring linear distance and angles which are formed between lines produced through body markers and horizontal or vertical lines on digital photographs by using software specifically designed for this purpose. The process quantifies postural changes through the application of photogrammetric principles to photographic images obtained during body movements. It involves the use of a digital camera and patients are encouraged to maintain their natural postures and movements as photographs are taken whilst acknowledging specific body landmarks with or without the use of plumblines ^107, 123, 231, 125, 129, 156, 157, 227– 230^	Yes ^123, 230^	Yes ^231^	Yes ^107, 123, 227^	No: Evidence not found.	No: Significant training required to use the tool. The tool can involve specialist equipment including image-based computer apps, reflective markers for anatomical reference points and camera ^107, 129, 157, 227– 231^	No: Minimal or no facilitation of clinician-patient interaction and collaboration through little or no feedback from the patient/active involvement of the patient in the assessment process ^107, 129, 156, 157, 227– 231^	Hard: Takes longer than 30 minutes to complete. Requires computer software application and interpretation ^107, 129, 157, 227– 231^	Postural-structural-biomechanical model of pain: Based on the assumption that there are specific correct and wrong postures ^107, 123, 231, 125, 129, 156, 157, 227– 230^
Electromagnetic measurement device	Electromagnetic measurement device is a wearable device that uses magneto-inertial measurement units to measure the angles of the spine and the body segments. They were developed and applied to solve the application problems of image-based methods of postural analysis. It is a three-dimensional measurement device that consists of a transmitter and receivers. A low-frequency magnetic field is generated by the transmitter and detected by the receivers. The positions and orientations of the receiver relative to the transmitter can be calculated by the system. These devices are often worn on the backs of patients around the T3, T12, and S1 vertebrae. The reference system used for validation is a stereophotogrammetric motion capture system. The measured variables for identifying the posture were the kyphosis and the lordosis angles, as well as the range of movement of the body segments. This system is wearable, inexpensive, and easy to set up in non-structured environments which makes it to have a wide applicability in posture evaluation and in clinical settings. ^131, 207, 232, 233^	No Insufficient evidence. ^234^	No: Evidence not found.	No Insufficient evidence. ^234^	No: Evidence not found.	No: Significant training required to use the tool. The tool involves specialist equipment including three-dimensional measurement device that consists of a transmitter and receivers that uses magneto-inertial measurement units to measure the angles of the spine and the body segments ^207, 232– 234^. ^131^	No: Minimal or no facilitation of clinician-patient interaction and collaboration through little or no active involvement of the patient in the assessment process ^207, 232– 234^. ^131^	Hard: Takes longer than 30 minutes to complete. Requires specialist equipment which can be influenced by the environment and signal extraction difficulties ^207, 232– 234^. ^131^	Postural-structural-biomechanical model of pain: Based on the assumption that there are specific correct and wrong postures ^207, 232– 234^. ^131^
ORFQ	The ORFQ is a 25-item self-report questionnaire of occupational biomechanical factors. The first five items measure work organisational factors such as work pressure and stress. The other items assess exposure to biomechanical factors such as bending, twisting, lifting, pulling, pushing, forceful movements and static postures like prolonged sitting, awkward postures and whole body vibrations. There is a first introductory question ‘ ***please describe the main tasks of your job’*** which is open, not numbered, and is not one of the 25 items in the questionnaire ^235, 236^	No: Evidence not found.	No: Evidence not found.	Yes ^235^	No: Evidence not found.	Yes: Little or no training is required to use the tool. No specialist equipment is required for the tool. ^235^	Yes: Significantly facilitates clinician-patient interaction and collaboration through requiring feedback from or active involvement of the patient in the assessment process. ^235^	Hard: Although the questionnaire can be completed within 30 minutes for literate people, the wording of the items and the response options have been difficult for people with low literacy to comprehend when interviewer administered ^237, 238^	Postural-structural-biomechanical model of pain: Based on the assumption that there are specific correct and wrong postures. ^235^
Computer based 3D bodyscanner/Computer based posture evaluation	This is a tool that uses advanced imaging technology to create a detailed three-dimensional representation of the body. It is used in anthropometry to quantify the morphology of the human body. In this system, a software is used to capture the volume of a person from whom a 3D avatar is created. The software is then used to mark specific landmarks on the body from which posture is extracted from the software. The scanner may also have a scale for balance metrics. 3D scans can be used to determine postural change by comparing scans before and after treatment. ^239– 241^	Yes ^242^	No: Evidence not found.	No: Evidence not found.	No: Evidence not found.	No: Significant training required to use the tool. The tool involves specialist equipment including image based computer software and 3D scanners. ^239– 241^	No: Minimal or no facilitation of clinician-patient interaction and collaboration through little or no feedback from the patient/active involvement of the patient in the assessment process. ^239– 241^	Hard: Can take longer than 30 minutes to complete. Requires computer software application and interpretation. ^239– 241^	Postural-structural-biomechanical model of pain: Based on the assumption that there are specific correct and wrong postures. ^239– 241^
4D formetric device ^153^	These are 4D spine and posture devices that contrary to simple 3D measurement methods which only detect individual measurement performs complete shape scanning using non-contact 4D scanning devices which is used to generate scan sequences of postures per second via a computer which produces the images. ^153^	Yes ^153, 243^	No ^244^	Yes ^153, 243^	Yes ^244^	No: Significant training required to use the tool. The tool involves specialist equipment including computers, computer softwares cameras, and 4D scanners ^153, 243^	No: Minimal or no facilitation of clinician-patient interaction and collaboration through little or no feedback from the involvement of the patient in the assessment process. ^153, 243^	Hard: Can take longer than 30 minutes to complete. Requires computer software application and interpretation. ^153, 243^	Postural-structural-biomechanical model of pain: Based on the assumption that there are specific correct and wrong postures. ^153, 243^
3D postural assessment with Global Posture System ^154^	The Global Posture System device is used to examine postural displacement variables. It has a unit for podoscopic analysis, a unit for postural analysis, and a stability measuring platform, and it comes with an image acquisition system and custom software. The camera of the image acquisition system was positioned 107cm from the ground and 190cm from the subject. The posture of the head in relation to the thoracic region was analysed in terms of translations and rotations. ^154^	Yes ^154^	No: Evidence not found.	Yes ^154^	No: Evidence not found.	No: Significant training required to use the tool. The tool involves specialist equipment including computers, computer softwares and cameras. ^154^	No: Minimal or no facilitation of clinician-patient interaction and collaboration through little or no feedback from the involvement of the patient in the assessment process. ^154^	Hard: Can take longer than 30 minutes to complete. Requires computer software application and interpretation. ^154^	Postural-structural-biomechanical model of pain: Based on the assumption that there are specific correct and wrong postures. ^154^
Postural monitor and feedback device ^245^	This is a device used to monitor and document lumbopelvic forward flexion posture which then provides audio feedback every time the user assumes a lumbopelvic forward bending posture that exceeds predefined thresholds. It is the wearable motion sensors within the device that allows the monitoring of lumbopelvic movement patterns and the provision of postural feedback during daily life and occupational activities. ^149, 205, 246, 247^	Yes ^245^	No: Evidence not found.	Yes ^245^	No: Evidence not found.	No: Although little or no training is required to use the device, there is a need for a postural monitor and feedback device for each patient. ^149, 205, 246, 247^	Yes: Significantly facilitates clinician-patient interaction and collaboration through requiring feedback from or active involvement of the patient in the assessment process. ^149, 205, 246, 247^	Easy: Can be completed within 30 minutes and it is easy to interpret. ^149, 205, 246, 247^	Postural-structural-biomechanical model of pain: Based on the assumption that there are specific correct and wrong postures. ^149, 205, 246, 247^
PostureScreen Mobile application. ^155^	This is a mobile application designed for the evaluation of static posture, particularly forward head. Patients stand with equal weight on both feet and photographs are taken from a distance of 10 feet and a height of 3.5 feet. Specific anatomical points determined for postural analysis are marked on the photographs in the mobile application. After determining the reference points, the application calculates lateral and anterior head tilt (in centimeters) using proprietary algorithms. ^155^	Yes. ^155, 248, 249^	No: Evidence not found.	Yes. ^155, 248, 249^	No: Evidence not found.	No: Although little or no training is required to use the application, there is a need for additional instrument such as a compatible smart phone and the application. ^155, 248, 249^	No: Minimal or no facilitation of clinician-patient interaction and collaboration through little or no feedback from the active involvement of the patient in the assessment process. ^155, 248, 249^	Hard: Although it can take shorter than 30 minutes to complete, it requires computer software application and interpretation. ^155, 248, 249^	Postural-structural-biomechanical model of pain: Based on the assumption that there are specific correct and wrong postures. ^155, 248, 249^
Instrumented insoles plus single chest-mounted accelerometer. ^126^	This device measured a combination of posture and spinal loading following the completion of the following tasks – lifting from the floor, overextended lifting, asymmetrical lifting, lifting from a height, lowering a weight, pushing and pulling, standing and walking. These activities were then analysed using Revised NIOSH (National Institute for Occupational Safety and Health – an ergonomic risk assessment tool for manual material handling tasks) Lifting Equation (RNLE) to demonstrate the ability of the machine learning classifier. Frequency Independent Recommended Weight Limit (FIRWL) was calculated initially, then used to calculate Recommended Weight Limit (RWL) and Lifting Index (LI) using classifier outputs for activity, load, and frequency. FIRWL was computed at the start and end of each lifting activity, with the minimum FIRWL considered as the recommended limit for the entire activity. ^126^	Yes. ^126^	No: Evidence not found.	Yes. ^126^	No: Evidence not found.	No: Significant training required to use the tool. The tool involves specialist equipment including special insoles and accelerometers. ^126^	No: Minimal or no facilitation of clinician-patient interaction and collaboration through little or no feedback from the active involvement of the patient in the assessment process. ^126^	Hard: Can take longer than 30 minutes to complete. Requires complex mathematical equation and software application and interpretation. ^126^	Postural-structural-biomechanical model of pain: Based on the assumption that there are specific correct and wrong postures. ^126^
Q-BAPHYP. ^128^	Q-BAPHYP uses language which is accessible to teenagers, and is formed by 35 closed questions (Likert scale) divided into 4 dimensions, grouped according to the postural habits and location: in the classroom (sitting 8 questions; standing 2 questions; body movement 1 questions), at home (sitting – 8 questions; body movement 1 question; standing 2 questions; position when watching TV 3 questions), carrying and lifting objects (backpack 2 questions, from the floor 2 questions) and teachers’ disciplinary guidance in relation to the student’s body position in the classroom (3 questions). The average time for completion was seven minutes. There were 5 alternative answers to each item: never, hardly ever, often, always, do not know/remember. The Likert scale used in this questionnaire was bipolar, for positive statements (good postural habits) the score starts at -2 (never) and goes up to 2 (always), while in the negative ones, it starts at 2 and provides scores with a sum of points. Positive scores suggest that the individual is aware of proper postural habits whereas negative scores indicate unsuitable habits. These score values help the healthcare professionals to identify the young person’s awareness of the postural habit adopted. ^128^	No: Evidence not found.	No: Evidence not found.	Yes ^250^	No: Evidence not found.	Yes Little or no training is required to use the tool. No specialist equipment is required for the tool. ^128, 250^	Yes: Significantly facilitates clinician-patient interaction and collaboration through requiring feedback from or active involvement of the patient in the assessment process. ^128, 250^	Easy: Can be completed within 30 minutes and it is easy to interpret. ^128, 250^	Postural-structural-biomechanical model of pain: Based on the assumption that there are specific correct and wrong postures. ^128, 250^
CROM device ^132^	The CROM device is allowed to rest comfortably around the participant’s head while resting above the ear whilst assuming a sitting position in a backed chair with hands relaxed on the lap, with hips and knees at approximately 90 degrees, and weight equally distributed on the seat. The CROM instrument is aligned over the bridge of the nose and ears and the Velcro straps are fastened and located posteriorly to secure the device to the head. The forward head arm is attached to the instrument at the bridge of the nose. The participants head is then positioned so that the sagittal dial meter read zero to achieve horizontal placement of the head and ensure that the eyes are directed straight ahead. The same researcher palpates the C7 spinous process and places the inferior foot of the vertebra locator on the C7 spinous process. The vertical alignment of the vertebra locator is ensured using the bubble level, adjusting until the bubble on the superior head is within the marked center position. Participants are instructed to keep the eyes looking straight ahead and to protrude and retract the lower cervical spine 3 times. After performing this movement pattern, the participant is told to “allow your head to assume its most comfortable resting position.” The measurement is recorded in 0.5 cm increments representing the point reached at the 90-degree intersection of the vertebra locator and the forward head arm. 3 trials are recorded for each participant, and the average of the results are calculated. ^132^	Yes ^251, 252^	No: Evidence not found.	Yes ^252, 253^	No: Evidence not found.	Yes ^252, 253^	No Minimal or no facilitation of clinician-patient interaction and collaboration through little or no feedback from the active involvement of the patient in the assessment process. ^132, 251– 253^	Easy Can be completed within 30 minutes and it is easy to interpret. ^132, 251– 253^	Postural-structural-biomechanical model of pain: Based on the assumption that there are specific correct and wrong postures. ^132, 251– 253^
Video/video recording	This entails video recording the patient’s movement and posture following which a detailed postural analysis is performed. Video recording is often combined with other observational methods, questionnaires or device-based methods of postural assessment. ^114, 165, 182– 191, 166, 194, 222, 167– 170, 178, 180, 181^	Yes. ^114, 165, 182– 191, 166, 194, 222, 167– 170, 178, 180, 181^	No: Evidence not found.	Yes. ^114, 165, 182– 191, 166, 194, 222, 167– 170, 178, 180, 181^	No: Evidence not found.	No: Although little or no training is required to use a video machine, there is a need for subsequent detailed postural analysis. ^114, 165, 182– 191, 166, 194, 222, 167– 170, 178, 180, 181^	No: Minimal or no facilitation of clinician-patient interaction and collaboration through little or no feedback from the patient/active involvement of the patient in the assessment process. ^114, 165, 182– 191, 166, 194, 222, 167– 170, 178, 180, 181^	Hard: Can take longer than 30 minutes to complete depending on which body segment(s) is being analysed. May require additional component of assessment using another tool. ^114, 165, 182– 191, 166, 194, 222, 167– 170, 178, 180, 181^	Postural-structural-biomechanical model of pain: Based on the assumption that there are specific correct and wrong postures. ^114, 165, 182– 191, 166, 194, 222, 167– 170, 178, 180, 181^
Self-developed questionnaire item(s)/surveys (specific for the different studies)	Self-developed questionnaire item(s) were specifically developed for each of the studies. They mainly assessed knowledge about basic ergonomics, spine anatomy and spine biomechanics, correct and incorrect postures during daily functional and occupational activities ^11, 81, 138– 143, 146, 147, 152, 85, 100, 115, 119, 134– 137^	No: Evidence not found.	No: Evidence not found.	No: Evidence not found.	No: Evidence not found.	Yes: Little or no training is required to use the questionnaires. No specialist equipment is required for the questionnaires ^11, 81, 138– 143, 146, 147, 152, 85, 100, 115, 119, 134– 137^	Yes: Significantly facilitates clinician-patient interaction and collaboration through requiring feedback from or active involvement of the patient in completing the questionnaires ^11, 81, 138– 143, 146, 147, 152, 85, 100, 115, 119, 134– 137^	Easy: Can be completed within 30 minutes and they are easy to interpret ^11, 81, 138– 143, 146, 147, 152, 85, 100, 115, 119, 134– 137^	Postural-structural-biomechanical model of pain: Based on the assumption that there are specific correct and wrong postures. ^11, 81, 138– 143, 146, 147, 152, 85, 100, 115, 119, 134– 137^
Self-reports (from participants)	These involved participants verbally describing the postures they adopted with or without demonstration of those postures as well as newly learnt correct postures and exercises to the recording practitioner. ^86, 145^	No: Evidence not found.	No: Evidence not found.	No: Evidence not found.	No: Evidence not found.	Yes: Little or no training is required. No specialist equipment is required. ^86, 145^	Yes: Significantly facilitates clinician-patient interaction and collaboration through requiring feedback from or active involvement of the patient in the assessment process. ^86, 145^	Easy: Can be completed within 30 minutes and they are easy to interpret. ^86, 145^	Postural-structural-biomechanical model of pain: Based on the assumption that there are specific correct and wrong postures. ^86, 145^
Diary/self-developed diary sheets/Postural hygiene booklet	These are used to track changes in posture over time and to track the effectiveness of interventions. These diaries usually contain measurements taken, observations made, record of interventions administered/received, and can be used to convey information to other healthcare professionals regarding the patient’s posture. ^148, 151, 254^	No: Evidence not found.	No: Evidence not found.	No: Evidence not found.	No: Evidence not found.	Yes Little or no training is required. No specialist equipment is required. ^148, 254^	Yes: Significantly facilitates clinician-patient interaction and collaboration through requiring feedback from or active involvement of the patient in the assessment process. ^148, 151, 254^	Easy: Can be completed within 30 minutes and they are easy to interpret. ^148, 151, 254^	Postural-structural-biomechanical model of pain: Based on the assumption that there are specific correct and wrong postures. ^148, 151, 254^
Demonstration by clinician or patient	This involved the demonstration of incorrect and correct postures by the patient or clinician to demonstrate current postural habits, and improvement in postural habits following an intervention. ^145^	No: Evidence not found.	No: Evidence not found.	No: Evidence not found.	No: Evidence not found.	Yes: Little or no training is required. No specialist equipment is required. ^145^	Yes: Significantly facilitates clinician-patient interaction and collaboration through requiring feedback from or active involvement of the patient in the assessment process. ^145^	Easy: Can be completed within 30 minutes and they are easy to interpret. ^145^	Postural-structural-biomechanical model of pain: Based on the assumption that there are specific correct and wrong postures. ^145^
Observation by the clinician or researcher	This entails the observation of patients in their workplaces by the practitioner, and was often combined with the standardised questionnaire and worksheet based postural outcome measures. ^114, 165, 178, 181– 188, 192, 166, 193– 195, 222, 171– 177^	No: Evidence not found.	No: Evidence not found.	No: Evidence not found.	No: Evidence not found.	Yes Little or no training is required. No specialist equipment is required.	No: Minimal or no facilitation of clinician-patient interaction and collaboration through little or no feedback from the patient/active involvement of the patient in the assessment process. ^114, 165, 178, 181– 188, 192, 166, 193– 195, 222, 171– 177^	Hard: Can take longer than 30 minutes to complete and requires observation in the workplace. ^114, 165, 178, 181– 188, 192, 166, 193– 195, 222, 171– 177^	Postural-structural-biomechanical model of pain: Based on the assumption that there are specific correct and wrong postures. ^114, 165, 178, 181– 188, 192, 166, 193– 195, 222, 171– 177^

DIPA= Digital Imaged-based Postural Assessment; BackPEI: Back Pain and Body Posture Evaluation Instrument questionnaire; RULA: Rapid upper limb assessment; QEC: Quick Exposure Check; REBA: Rapid entire body assessment; OWAS: Ovako working posture analysis system; NERPA: novel ergonomic postural assessment method; PATH: posture, activity tools and handling measurement method; PAI: Posture assessment instrument; CUELA system: computer based measurement and long term analysis of stress upon musculoskeletal system; ALLA= Agricultural lower limb assessment; BEHALVES, Back-hEalthrelated postural Habits in dAiLy actiVitiES; ORFQ: Occupational risk factor questionnaire; Q-BAPHYP: Questionnaire of Body Awareness and Postural Habits of Young People; CROM device: Cervical range of motion device

## Discussion

This is the first study to explore the clinical utility of outcome tools for measuring exposure to postural risk factors for back and neck pain clinical outcomes. Findings from this study suggest that existing postural outcome tools have low clinical utility for back and neck pain. Majority of the tools were sophisticated computer-based or electronic devices, complex time-consuming questionnaires, with limited utility in non-occupational settings. The objective instrument-based postural measures can hardly be utilized outside of a laboratory setting and mostly measure posture under artificial conditions. ^36^ The easy-to-use tools were mostly unvalidated. The most achieved clinical utility domains were construct validity and inter/intra-rater reliability, and the least achieved domains were tools being underpinned by the biopsychosocial model of pain, ^40– 43^ sensitivity to clinical change, and predictive validity.

Postural outcome tools that are based on the postural-structural-biomechanical model of pain have the underlying assumption that there are universal ‘good’ and ‘bad’ postures for back and neck pain. Therefore, individuals whose daily activities involve these postures may develop apprehension regarding those activities which may lead to the development of fear avoidance beliefs. The subsequent fear avoidance behaviours that result from this is associated with adverse back and neck pain clinical outcomes. ^16^ The fear avoidance model explains how individuals avoid activities believed to cause pain, even when these activities are neither harmful nor painful, which can lead to disuse, deconditioning, and disability. Furthermore, fear avoidance beliefs may be associated with hypervigilance, and anticipation of pain during those activities of daily life in the postures understood as ‘incorrect’, which may in turn lead to muscle guarding and co-contraction, increasing pain and disability. ^18^ It is possible that this might be counteracted by employing postural outcome measures that are based on the biopsychosocial model of spinal pain. Postural outcome tools based on the biopsychosocial pain model might enable individuals to understand that posture may not be an aggravating factor for everyone, and that those for whom posture is an aggravating factor, it may be different in different people, and can be driven by cognitive, emotional, psychological, behavioural, physical, and social factors which may interact to perpetuate pain. Measuring posture within a biopsychosocial pain model may be associated with several potential benefits. For instance, it might prevent the development of fear avoidance behaviour and subsequent disability. Postural outcome measures based on the biopsychosocial model may also support active self-management by encouraging people to identify their individual aggravating postures, the potential biopsychosocial factors driving the aggravating posture, how to actively modify the aggravating postures and the factors driving them, identification of relieving postures, and the need to keep moving by regularly changing posture. Using postural outcome tools based on the biopsychosocial model aligns with the biopsychosocial pain model ^40– 43^ and the evidence-based recommendations for symptomatic relief of spinal pain episodes to prevent participation restrictions and disability. ^44– 46^ A biopsychosocial approach in the assessment and management of spinal pain enables the identification of multiple interacting factors affecting the patient, and the management of the patient on multiple levels to target those factors, their interactions, and the impact of their interactions. Previous belief that poor posture can cause or aggravate back and neck pain through increased stress on the muscles, ligaments, and joints with good posture protecting the spine by decreasing stress on these structures, keeping the joints in alignment, and improving efficiency of the musculoskeletal system is simplistic at best. This is because important factors such as biological vulnerability, physical activity and fitness levels, duration of sustained posture, spinal loading, and other biopsychosocial factors are not taken into consideration. Moreover, many ergonomic postural tools overestimate the risk of injuries, with some tools almost always predicting risk every time that they have been used. ^182^ Our recently completed but yet unpublished mixed-methods study suggests that many global spinal pain experts no longer believe in specific ‘good’ or ‘bad’ postures. However, a significant number of clinicians globally, and many spinal pain researchers in low and middle-income countries, may still believe in specific poor and good postures as risk factors for spinal pain and support their assessment and targeting. ^255^ This belief is also held by ergonomic engineers from whom a significant number of the studies and outcome tools included in this review were produced.

The fact that only two, and five measures had documented evidence of sensitivity to clinical change and predictive validity respectively, could explain the conflicting evidence regarding the effectiveness of interventions with a postural component. ^33– 35^ Furthermore, the lack of sensibility, easy format, and ease of use in clinical settings by a significant number of the tools could explain why many studies that investigated the effectiveness of postural interventions or complex interventions with a postural component in clinical settings either did not measure posture or used self-developed easy-to-use but non-validated tools. Therefore, these studies could not link the reported clinical benefits of the interventions to postural changes. ^26– 31, 35, 256– 261^ Another factor that could limit the applicability of the identified postural outcome measures is the restriction of measurement to only work-related risk factors. These tools did not acknowledge home related and other risk factors associated with daily living.

This scoping review has some limitations. Studies published in other languages apart from English, or in non-indexed journals, or studies that were not yet published at the time of this study might have contained more clinically useful postural outcome tools which were not included in this review. Another limitation is the possibility that some of the postural outcome tools which had low clinical utility may inherently have some of the defining features of clinical utility such as sensitivity to change, predictive validity etc., but these clinical utility domains had not been assessed in published studies at the time of this study. Finally, it is possible that the definition of ease of use of tools in clinical settings which was informed by the published literature and clinical experience might have been too stringent, thereby excluding potentially clinically useful postural outcome tools. However, this is unlikely since time consuming and highly technical tools may be difficult to implement in busy clinical settings.

### Implications of findings on clinical practice and future research

Evidence-based, simple, and clinically useful postural outcome tools that are based on the biopsychosocial pain model may be able to identify patterns of aggravating postures for different individuals as well as potential interaction of these postures with psychosocial and biomedical factors, and the impact of these interactions. This may be particularly useful in clinical settings as such short, simple, and easy to administer outcome tools will help to determine the multiple levels of factors that should be targeted by interventions. Furthermore, postural outcome tools based on the biopsychosocial pain model can be used in future epidemiological studies to clarify the relative contribution of postural factors to first onset of back and neck pain in different individuals. In addition, these tools may reduce the incidence, prevalence, and impact of fear avoidance beliefs due to the use of the current tools based on the postural-structural-biomechanical model of pain. Finally, new tools that are based on the biopsychosocial pain model may be useful in future clinical trials for isolating the impact of postural risk factors on clinical outcomes relative to potential interacting factors, and for understanding the mechanisms of action of clinical interventions.

## Conclusions

Existing postural outcome tools have limited clinical utility for back and neck pain. The domains of clinical utility reflected in studies in a decreasing order of achievement was construct validity and inter and intra-rater reliability (24 tools each), followed by sensibility (16 tools), ease of use in clinical settings (13 tools), easy format (13 tools), predictive validity (5 tools), sensitivity to change (2 tools), and underpinned by a biopsychosocial model of pain (no tool). Identified tools were mostly sophisticated computer-based or electronic devices; non-validated or complex time-consuming questionnaires; had limited predictive validity, sensitivity to change, and applicability in non-occupational settings; and were based on a postural-structural-biomechanical model of pain.

### Ethical statement

There are no ethical considerations for this study that utilised data from published studies.

## Data Availability

No data are associated with this article. Figshare: Exploring the clinical utility of postural outcome tools for back and neck pain clinical outcomes: a systematic scoping review, DOI: https://doi.org/10.6084/m9.figshare.28054454.v1 ^69^ The project contains the following reporting guidelines:
• Appendix 1 Search Strategy Postural Outcome tools F1000Research Data are available under the terms of the Creative Commons Zero “No rights reserved” data waiver (CC0 1.0 Public domain dedication). Figshare: Exploring the clinical utility of postural outcome tools for back and neck pain clinical outcomes: a systematic scoping review, DOI: https://doi.org/10.6084/m9.figshare.28054454.v1 ^69^ The project contains the following reporting guidelines:
• PRISMA-ScR-Fillable-Checklist COMPLETED_10Sept2019.docx Data are available under the terms of the Creative Commons Zero “No rights reserved” data waiver (CC0 1.0 Public domain dedication).
