# Supplementary material for: Exploring the clinical utility of postural outcome tools for back and neck pain clinical outcomes: a systematic scoping review
Source: F1000Res. 2025 Oct 15;14:44. Originally published 2025 Jan 8. [Version 2] doi: 10.12688/f1000research.160172.2 (PMC12603532; doi:10.12688/f1000research.160172.2)
Supplement: Peer review history

# Reviewer report: Reviewer response for version 2

**DOI:** 10.5256/f1000research.189647.r424443

**Published:** 2025-11-10

**Authors:** Stephen Sunday Ede

**Affiliations:** 1 University of Central Lancashire, Preston, UK

I have now read the revision, and I am happy to recommend accepting the paper for indexing.

Are the rationale for, and objectives of, the Systematic Review clearly stated?

Yes

Is the statistical analysis and its interpretation appropriate?

Yes

If this is a Living Systematic Review, is the ‘living’ method appropriate and is the search schedule clearly defined and justified? (‘Living Systematic Review’ or a variation of this term should be included in the title.)

Not applicable

Are sufficient details of the methods and analysis provided to allow replication by others?

Yes

Are the conclusions drawn adequately supported by the results presented in the review?

Yes

Reviewer Expertise:

Occupational health and safety

I confirm that I have read this submission and believe that I have an appropriate level of expertise to confirm that it is of an acceptable scientific standard.

# Reviewer report: Reviewer response for version 2

**DOI:** 10.5256/f1000research.189647.r424442

**Published:** 2025-11-07

**Authors:** Saturday Nicholas Oghumu

**Affiliations:** 1 Physiotherapy, University of Benin School of Basic Medical Science, Benin City, Edo, Nigeria

Authors have diligently responded to my comments, Well done to authors, I have no further comments.

Are the rationale for, and objectives of, the Systematic Review clearly stated?

Yes

Is the statistical analysis and its interpretation appropriate?

Not applicable

If this is a Living Systematic Review, is the ‘living’ method appropriate and is the search schedule clearly defined and justified? (‘Living Systematic Review’ or a variation of this term should be included in the title.)

Not applicable

Are sufficient details of the methods and analysis provided to allow replication by others?

Yes

Are the conclusions drawn adequately supported by the results presented in the review?

Partly

Reviewer Expertise:

Pain medicine, orthopedics, sport medicine, physiotherapy, rehabilitation, exercise medicine

I confirm that I have read this submission and believe that I have an appropriate level of expertise to confirm that it is of an acceptable scientific standard.

# Reviewer report: Reviewer response for version 1

**DOI:** 10.5256/f1000research.176017.r405084

**Published:** 2025-09-10

**Authors:** Stephen Sunday Ede

**Affiliations:** 1 University of Central Lancashire, Preston, UK

Dear authours,

 I had the opportunity to review your manuscript that is focused on exploring the clinical utility of postural outcome tools for back and neck pain clinical outcomes using the scoping review methodology. The study methodology demonstrated good rigour and clarity and contributed relevant discussion to the literature. Nevertheless, we hope these comments will identify some ways the manuscript can be enhanced to improve its contribution. 

**Introduction**

 The paper is currently long (more like a dissertation format) and can be reduced by consolidating the writing. For instance, in the inclusion criteria, it can be more brief and focus on the criteria that were used. Settings were not used and do not necessarily need to be discussed. 

**Result**

 Table 1 and Table 2 both require a lengthy read, given that these are summaries of already published information. Could the present work possibly focus on presenting the identified postural outcome tools, their clinical utility, etc, which is the objective of the present study? 

**Discussion**

 The proposal for a postural outcome tool that is based on the biopsychosocial pain model is rather speculative, as it does not point to specific examples or instances. While pain can be described in a biopsychosocial model, posture seemed rather a biomedical construct, which would explain your findings of lack of postural measures with this clinometry. Giving an example of what is meant might help drive home the clinical implications of the findings.

Are the rationale for, and objectives of, the Systematic Review clearly stated?

Yes

Is the statistical analysis and its interpretation appropriate?

Yes

If this is a Living Systematic Review, is the ‘living’ method appropriate and is the search schedule clearly defined and justified? (‘Living Systematic Review’ or a variation of this term should be included in the title.)

Not applicable

Are sufficient details of the methods and analysis provided to allow replication by others?

Yes

Are the conclusions drawn adequately supported by the results presented in the review?

Yes

Reviewer Expertise:

Occupational health and safety

I confirm that I have read this submission and believe that I have an appropriate level of expertise to confirm that it is of an acceptable scientific standard, however I have significant reservations, as outlined above.

# Response

**Published:** 2025-10-12

**Authors:** Chinonso Igwesi-Chidobe

**Affiliations:** Global Population Health, University of Nigeria, Enugu, Enugu, Nigeria

**Response to reviewer 2 comments**

1. Introduction

I have now reduced the word count of the manuscript overall by making different sections more succinct. However, I have also had to add additional detail required by other reviewers. It was important to retain the inclusion criteria suggesting that all contexts were eligible to clarify that we did not want to restrict this study to only occupational or clinical settings. All changes are tracked.

  

1. Result

Table 2 answers the key objective of this study as it focuses on the clinical utility of the postural outcome tools. Table 1 is important as it gives context to the findings in Table 2. For instance, countries where tools are used, population and study design characteristics etc are provided in one place for readers who may be interested in this. Therefore, I have retained Tables 1 and 2 as some readers may choose not to read these in detail while important summaries are available in one place for readers who require more detail.

  

1. Discussion

I have now added details and examples on how postural outcome assessment can be assessed within the biomedical and biopsychosocial models in both the introduction and discussion sections. Specifically, I have added the following statements to the Introduction section: ‘The biomedical pain model assumes that pain is a result of biological and mechanical factors including structural changes from tissue damage. This model emphasizes anatomical and pathological causes of pain and assumes a direct relationship between pain and structural changes. In contrast, pain is viewed as a complex interaction between biological, psychological, and social factors within the biopsychosocial pain module which align with current research evidence. We propose that to contextualise postural outcome tools in relation to the biomedical or biopsychosocial pain models, tools that measure specific ‘right’ and ‘wrong’ postures reflect a biomedical orientation while tools that measure posture in terms of functional and subjective relevance to different people rather than as specific ‘correct’ and ‘incorrect’ postures across all individuals reflect a biopsychosocial orientation in line with the literature. We state that tools that measure posture as personal, individualised and context dependent measures the biological construct but does this within the biopsychosocial pain model rather than the biomedical pain model.

 As research evidence suggests that there is no universal ideal posture that can either prevent or reduce back and neck pain and the associated clinical outcomes, postural outcome tools underpinned by the biomedical pain model are unlikely to explain most back and neck pain. This can explain conflicting research findings previously discussed. In contrast, postural outcome tools that align with the biopsychosocial pain model would measure posture as personalised taking into consideration different personal and social contexts within which the measurement was taken. For instance, questionnaires that assess personal aggravating and relieving postures and in what personal (e.g., fears, beliefs) and social (home, school or work environments) contexts these postures occur; functional and performance-based assessments that observe posture during functional tasks to identify the movement patterns that are associated with pain, and whether there is ‘guarding’ due to fear or beliefs during specific movements without assumption of any universal ‘correct/right’ and ‘incorrect/wrong’postures may be typical examples of postural measurement within the biopsychosocial model. Measurement in this way acknowledges that although posture falls within the biological component of the biopsychosocial pain model, its relevance and therefore its measurement needs to happen within the biopsychosocial model’.

 And added the following statements to the discussion section: ‘No universal ideal posture has been identified for preventing or reducing back and neck pain. This suggests that postural outcome tools underpinned by the biomedical pain model with the assumption of specific ‘right’ and ‘wrong’ postures for all people will have limited clinical applicability. A biopsychosocial approach to postural measurement may offer an evidence-based and more nuanced substitute. Using the biopsychosocial pain model, posture can be measured as an individualised construct shaped by personal and contextual factors with assessments aiming to identify personal aggravating and relieving postures for different individuals and under what personal (e.g., fears, beliefs) and social (e.g., home, school or work environments) contexts these postures occur’.

# Reviewer report: Reviewer response for version 1

**DOI:** 10.5256/f1000research.176017.r359811

**Published:** 2025-01-27

**Authors:** Saturday Nicholas Oghumu

**Affiliations:** 1 Physiotherapy, University of Benin School of Basic Medical Science, Benin City, Edo, Nigeria

Dear Editor, it is obvious that the authors of this manuscript have dedicated a great deal of effort in writing this scoping review on clinical utility of postural outcome tools for back and neck pain clinical outcomes. It is also commendable to say that the authors have carefully follow the methodology for conducting a systematic review and provided details with reference for modification to scoping review. However, the below comments are my submissions for authors consideration.

**Abstract**

 The clause “Tools being underpinned by the biopsychosocial model of pain” may not be necessary here given that it is unrelated to the title, aim and cannot be accounted for by the articles reviewed in both the methodology and results of the study.

**Introduction**

 Although, I agree with the authors that “posture may not even be an aggravating factor in some people” authors inclination in aligning evidence-based postural outcome tools with evidence-based biopsychosocial model of spinal pain is not backed up with facts. What construct of the biopsychosocial (biologic, psychosocial & social) model of spinal pain does posture defines? Authors should provide information regarding how the combination of these constructs of biopsychocial model of spinal pain interplay in the complexity and multidimensional nature of spinal pain, as well as their corresponding clinical outcomes  for the understanding of readership.

**Method**

 Inclusion criteria: The inclusion of studies of human participants with spinal pain of all ages may be too broad given the choice of mechanical pain. What influence does the participants age have in defining the mechanical nature of their pain? Authors should clarify, especially in the study limitation given the exclusion criteria of spinal pain of systemic pathology which may be unnoticeable in children with spinal pain? Does this in any way affect the outcome of the postural outcome tools in studies with these age categories? This should be discussed in the discussion section.

**Results**

 Majority of the postural outcome tools assessed are patient reported outcome measures, but the minimal clinically important difference (MCID) of these tools were not accounted for. Why?

**Discussion**

 Provide clarity of postural outcome tools based on the biopsychosocial pain model. Are you implying that postural outcome tools should reflect the psychosocial components of the biopsychosocial model apart from assessing posture? Then briefly provide additional details of this possibility.

 Thank you.

Are the rationale for, and objectives of, the Systematic Review clearly stated?

Yes

Is the statistical analysis and its interpretation appropriate?

Not applicable

If this is a Living Systematic Review, is the ‘living’ method appropriate and is the search schedule clearly defined and justified? (‘Living Systematic Review’ or a variation of this term should be included in the title.)

Not applicable

Are sufficient details of the methods and analysis provided to allow replication by others?

Yes

Are the conclusions drawn adequately supported by the results presented in the review?

Partly

Reviewer Expertise:

Pain medicine, orthopedics, sport medicine, physiotherapy, rehabilitation, exercise medicine

I confirm that I have read this submission and believe that I have an appropriate level of expertise to confirm that it is of an acceptable scientific standard, however I have significant reservations, as outlined above.

# Response

**Published:** 2025-10-12

**Authors:** Chinonso Igwesi-Chidobe

**Affiliations:** Global Population Health, University of Nigeria, Enugu, Enugu, Nigeria

**Response to reviewer 1 comments**

1. Abstract

I have modified the clause “Tools being underpinned by the biopsychosocial model of pain” to ‘Alignment with the biopsychosocial pain model’ and modified aspects of the abstract including statements ‘Clinical utility was defined by psychometric and clinimetric criteria including construct validity, predictive validity, intra and inter rater reliability, sensitivity to change, ease of use, sensibility, format, and alignment with the biopsychosocial pain model’ which ensures alignment with the study title, and aim.

 Furthermore, I have included additional statements ‘The biomedical pain model assumes that pain is a result of biological and mechanical factors including structural changes from tissue damage. This model emphasizes anatomical and pathological causes of pain and assumes a direct relationship between pain and structural changes. In contrast, pain is viewed as a complex interaction between biological, psychological, and social factors within the biopsychosocial pain module which align with current research evidence.

 To contextualise postural outcome tools in relation to the biomedical and biopsychosocial pain models, tools that measure specific ‘right’ and ‘wrong’ postures reflect a biomedical orientation while tools that measure posture in terms of functional and subjective relevance to different people rather than as specific ‘correct’ and ‘incorrect’ postures across all individuals reflect a biopsychosocial orientation. Specifically, tools that measure posture as personal, individualised and context dependent align with the biopsychosocial pain model.

 We state that postural outcome tools underpinned by the biomedical pain model are unlikely to explain most back and neck pain as research evidence suggests that there is no universal ideal posture that can either prevent or reduce back and neck pain and the associated clinical outcomes. In contrast, postural outcome tools that align with the biopsychosocial pain model would measure posture as personalised assessment taking into consideration different personal and social contexts within which the measurement was taken. Questionnaires that assess personal aggravating and relieving postures and in what personal (e.g., fears, beliefs) and social (home, school or work environments) contexts these postures occur; functional and performance-based assessments that observe posture during functional tasks to identify the movement patterns that are associated with pain, and whether there is ‘guarding’ due to fear or beliefs during specific movements without assumption of any universal ‘correct/right’ and ‘incorrect/wrong’postures are possible examples.

 Therefore, this systematic scoping review aimed to map and summarise the clinical utility (construct validity, predictive validity, intra and inter rater reliability, sensitivity to change, ease of use in clinical settings, sensibility, format of the tool, and alignment with the biopsychosocial pain model) of existing outcome tools for measuring exposure to postural risk factors for back and neck pain clinical outcomes. These clinical outcomes included but were not limited to first onset of back and/or neck pain, chronicity/perpetuation of symptoms, pain including the nature and intensity, disability etc.’ was added to the introduction,

 ‘Specifically, we explored the content of each postural outcome tool to determine whether they measured specific ‘right’ and ‘wrong’ postures which will not reflect a biopsychosocial orientation, or measured posture as personalised assessment taking into consideration different personal and social contexts within which the measurement was taken, including identification of personal aggravating and relieving postures and in what personal and social contexts they occur as previously described’ was added to the methods section, and ‘…..’ was added to the results sections to explain how alignment with the biopsychosocial pain model can be answered with data from the articles reviewed. All changes are tracked in the resubmission.

 2. Introduction

 I have modified the introduction section to clarify the construct that posture captures within the biopsychosocial model and provided detailed information regarding its measurement within the biopsychosocial model with examples. These are reflected in these statements that I added to the introduction section ‘The biomedical pain model assumes that pain is a result of biological and mechanical factors including structural changes from tissue damage. This model emphasizes anatomical and pathological causes of pain and assumes a direct relationship between pain and structural changes. In contrast, pain is viewed as a complex interaction between biological, psychological, and social factors within the biopsychosocial pain module which align with current research evidence.

 To contextualise postural outcome tools in relation to the biomedical and biopsychosocial pain models, tools that measure specific ‘right’ and ‘wrong’ postures reflect a biomedical orientation while tools that measure posture in terms of functional and subjective relevance to different people rather than as specific ‘correct’ and ‘incorrect’ postures across all individuals reflect a biopsychosocial orientation. Specifically, tools that measure posture as personal, individualised and context dependent align with the biopsychosocial pain model.

 We state that postural outcome tools underpinned by the biomedical pain model are unlikely to explain most back and neck pain as research evidence suggests that there is no universal ideal posture that can either prevent or reduce back and neck pain and the associated clinical outcomes. In contrast, postural outcome tools that align with the biopsychosocial pain model would measure posture as personalised assessment taking into consideration different personal and social contexts within which the measurement was taken. Questionnaires that assess personal aggravating and relieving postures and in what personal (e.g., fears, beliefs) and social (home, school or work environments) contexts these postures occur; functional and performance-based assessments that observe posture during functional tasks to identify the movement patterns that are associated with pain, and whether there is ‘guarding’ due to fear or beliefs during specific movements without assumption of any universal ‘correct/right’ and ‘incorrect/wrong’postures are possible examples. Measurement in this way acknowledges that although posture falls within the biological component of the biopsychosocial pain model, its relevance and therefore its clinical assessment needs to adopt the biopsychosocial model. Therefore, this systematic scoping review aimed to map and summarise the clinical utility (construct validity, predictive validity, intra and inter rater reliability, sensitivity to change, ease of use in clinical settings, sensibility, format of the tool, and alignment with the biopsychosocial pain model) of existing outcome tools for measuring exposure to postural risk factors for back and neck pain clinical outcomes.’. All changes are tracked.

 3. Method

 In response to the reviewer’s comment, in the inclusion criteria, I have now included the statement ‘Age was not restricted to adults due to evidence suggesting that mechanical spinal pain in children and adolescents may be underestimated.14,15’ In addition, in the limitations paragraph of the discussion section, I have now included the statements ‘The nature, severity, prevalence and impact of spinal pain are likely to increase with increasing age, and non-specific mechanical spinal pain is more likely in adults suggesting that the findings of this study may have better applicability in adults. This potential limitation is ameliorated by the fact that some included postural outcome tools were utilised among children with non-specific mechanical spinal pain’. All changes are tracked.

 4. Results

 I have now provided a justification for why minimal clinically important difference (MCID) of these tools were not used by including these statements in the methods section: “Minimal clinically important difference (MCID) was not included in clinical utility investigation because the measurement of posture in people with or at risk of spinal pain is a complex and objective construct with complex unpredictable relationships with pain. Furthermore, posture being more of a predictor variable than a primary or criterion variable, makes the assessment of MCID less relevant. In contrast, MCIDs are used to quantify meaningful changes in clinical outcomes that are important to patients such as reduced pain and disability, and improved function and quality of life.” All changes are tracked.

 5. Discussion

 I have now provided clarity of postural outcome tools based on the biopsychosocial pain model by including many statements to the introduction and discussion sections. These include: (1)‘The biomedical pain model assumes that pain is a result of biological and mechanical factors including structural changes from tissue damage. This model emphasizes anatomical and pathological causes of pain and assumes a direct relationship between pain and structural changes. In contrast, pain is viewed as a complex interaction between biological, psychological, and social factors within the biopsychosocial pain module which align with current research evidence. We propose that to contextualise postural outcome tools in relation to the biomedical or biopsychosocial pain models, tools that measure specific ‘right’ and ‘wrong’ postures reflect a biomedical orientation while tools that measure posture in terms of functional and subjective relevance to different people rather than as specific ‘correct’ and ‘incorrect’ postures across all individuals reflect a biopsychosocial orientation. We state that tools that measure posture as personal, individualised and context dependent measures a biological construct but does this within the biopsychosocial pain model rather than the biomedical pain model. As research evidence suggests that there is no universal ideal posture that can either prevent or reduce back and neck pain and the associated clinical outcomes, postural outcome tools underpinned by the biomedical pain model are unlikely to explain most back and neck pain. This can explain conflicting research findings previously discussed. In contrast, postural outcome tools that align with the biopsychosocial pain model would measure posture as personalised taking into consideration different personal and social contexts within which the measurement was taken. For instance, questionnaires that assess personal aggravating and relieving postures and in what personal (e.g., fears, beliefs) and social (home, school or work environments) contexts these postures occur; functional and performance-based assessments that observe posture during functional tasks to identify the movement patterns that are associated with pain, and whether there is ‘guarding’ due to fear or beliefs during specific movements without assumption of any universal ‘correct/right’ and ‘incorrect/wrong’postures may be typical examples of postural measurement within the biopsychosocial model. Measurement in this way acknowledges that although posture falls within the biological component of the biopsychosocial pain model, its relevance and therefore its measurement needs to happen within the biopsychosocial model.’ in the introduction section and (2)‘No universal ideal posture has been identified for preventing or reducing back and neck pain. This suggests that postural outcome tools underpinned by the biomedical pain model with the assumption of specific ‘right’ and ‘wrong’ postures will have limited clinical applicability. A biopsychosocial approach to postural measurement may offer a more nuanced alternative. Using the biopsychosocial pain model, posture can be measured as an individualised construct shaped by personal and contextual factors with assessments aiming to identify personal aggravating and relieving postures and under what personal (e.g., fears, beliefs) and social (home, school or work environments) contexts these postures occur. Postural outcome tools that are based on the postural-structural-biomechanical model (biomedical pain model) have the underlying assumption that there are universal ‘good’ and ‘bad’ postures for back and neck pain.’ in the discussion section. All changes are tracked.
